# Efficacy and tolerability of perampanel in patients with seizures in real-world clinical practice: A systematic review and meta-analysis

**DOI:** 10.3389/fphar.2023.1139514

**Published:** 2023-03-28

**Authors:** Liyan Hou, Jingjing Yang, Xuan Zhang, Na Li, Sheng Li, Lei Zhang, Jie Zhao, Qingshan Wang

**Affiliations:** ^1^ Dalian Medical University Library, Dalian Medical University, Dalian, China; ^2^ Department of Neurology, The First Affiliated Hospital of Dalian Medical University, Dalian, China; ^3^ National-Local Joint Engineering Research Center for Drug-Research and Development R & D of Neurodegenerative Diseases, Dalian Medical University, Dalian, China; ^4^ School of Public Health, Dalian Medical University, Dalian, China

**Keywords:** seizures, meta-analysis, real-world study, perampanel, systemic review

## Abstract

**Objectives:** The aim of this study was to systematically review the efficacy and tolerability of perampanel (PER) when used as add-on treatment or monotherapy in patients with epilepsy aged 12 years and older in routine clinical practice.

**Methods:** Electronic and clinical trials databases were searched for observational studies of PER published up to 1 March 2022. The outcomes of interest were responder rates, adverse effects (AEs), and withdrawal rates. Subgroup analyses were performed to explore the potential factors that might affect the efficacy and safety of PER usage.

**Results:** A total of 56 studies, which included 10,688 patients, were enrolled. The results showed that after 3, 6, and 12 months of PER treatment, the pooled 50% responder rates in patients with epilepsy were 50.0% (95% CI: 0.41–0.60), 44.0% (95% CI: 0.38–0.50), and 39.0% (95% CI: 0.31–0.48), respectively, and the pooled seizure-free rates were 24.0% (95% CI: 0.17–0.32), 21.0% (95% CI: 0.17–0.25), and 20.0% (95% CI: 0.16–0.24), respectively. Subgroup analyses revealed that the efficacy of PER could be affected by the way in which PER is administrated. Patients in the groups where PER was used as the first add-on, primary monotherapy, or combined with non–enzyme-inducing AEDs (non-EIAEDs) displayed a high 50% responder rate and seizure-free rate when compared with those in the late add-on, conversion therapy, or combined with the EIAEDs groups, respectively. Furthermore, the incidences of AEs at 3, 6, and 12 months of PER treatment were 46% (95% CI: 0.38–0.55), 52.0% (95% CI: 0.43–0.60), and 46.0% (95% CI: 0.40–0.52), respectively. The withdrawal rates due to AEs were 8.0% (95% CI: 0.06–0.11), 16.0% (95% CI: 0.13–0.20), and 16% (95% CI: 0.11–0.21) at 3, 6, and 12 months of PER treatment, respectively. Subgroup analyses showed a higher withdrawal rate in the rapid (30%, 95% CI: 0.22–0.38) than in the slow (12%, 95% CI: 0.06–0.18) titration group.

**Conclusion:** Altogether, PER was effective and could be fairly tolerated in both short-term and long-term usage in patients with epilepsy in routine clinical practice. Furthermore, PER appeared to be more effective when PER was used as the first add-on, monotherapy, or concomitant with non-EIAEDs.

**Systematic Review Registration**: https://www.crd.york.ac.uk/PROSPERO/, identifier CRD42022384532.

## Introduction

Epilepsy is a major mental and neurological disease and affects approximately 70 million people in the world (Nevitt et al., 2017; Lattanzi et al., 2022), accounting for approximately 5% of the total disability-adjusted life years for all neurological disorders (Begley et al., 2022). Despite various new therapeutic strategies being developed, such as responsive neurostimulation therapy, antiepileptic drugs (AEDs) are still the primary choice for epilepsy treatment in the clinic. However, up to 35% of newly diagnosed patients fail to achieve long-term remission with AED treatment (Janmohamed et al., 2020). Uncontrolled epilepsy could result in severe disability, psychosocial consequences, decreasing life quality, and increasing economic burden (Murray et al., 2012; Begley et al., 2022). It has been suggested that for patients who cannot obtain satisfactory seizure remission by the initial AEDs, receiving bitherapy combined with an AED of a different mechanism of action (MOA) could achieve seizure control and even make them seizure free (Hakami, 2021). Thus, it is urgent that novel AEDs are developed, especially for patients with refractory epilepsy and frequent comorbidities.

Common molecular targets of AEDs for the pharmacotherapy of epilepsy are on channels, GABA receptors, excitatory amino acids receptors, enzymes, and synaptic proteins (Lasoń et al., 2011). The α-amino-3-hydroxy-5-methyl-4-isoxazolepropionic acid (AMPA) receptor is critical in mediating rapid excitatory neurotransmission in the central nervous system and plays an important role in generating and spreading epileptic activity (Potschka and Trinka, 2019). Perampanel (PER), one of the third-generation antiseizure medications, is an oral, highly selective, and non-competitive antagonist of the AMPA receptor (Tsai et al., 2018). PER has already been approved for adjunctive treatment of partial-onset seizures (POSs), with or without secondarily generalized seizures in patients aged ≥12 years from more than 50 countries. Currently, for monotherapy in POS patients aged ≥12 years, PER has been licensed in the US and Japan only. Three phase III multi-centered, randomized controlled trials (RCTs) (Trials 304, 305, and 306) have demonstrated that PER is safe and efficacious as adjunctive treatment in patients aged over 12 years for refractory POS when compared with placebo (Krauss et al., 2012b; French et al., 2012; French et al., 2013), providing important information for the regulatory approval of PER. However, most RCT studies have focused on the efficacy of PER in patients with drug-resistant epilepsy; therefore, many patients with complex disorders and comorbidities (e.g., brain tumors, intellectual disability, and trauma) were excluded. In addition, in RCTs, the treatment regimens for PER could often not be personalized. Real-world evidence from observational studies could compensate for these drawbacks and recruit the “real-life” population with epilepsy who might not meet the inclusion criteria for RCTs. Therefore, real-world studies with PER have been gradually performed since 2016. However, these studies on the efficacy of PER in treating seizure-related outcomes have generated mixed results. For example, some studies found that patients using concomitant enzyme-inducing AEDs displayed lower clinical responses to PER than those receiving non–enzyme-inducing AEDs (Villanueva et al., 2016; Rinaldi and De Maria, 2018). However, some studies had reported similar responder rates to PER in patients with and without enzyme-inducing AEDs (Steinhoff et al., 2014; Youn et al., 2018).

Therefore, this meta-analysis study was designed and aimed to re-evaluate the efficacy and tolerability of PER, used as add-on treatment and monotherapy in patients with epilepsy aged 12 years and older using high-quality observational studies, which provides valuable insights for developers and prescribers in the routine clinical usage of PER.

## Methods

### Literature search strategy

Our meta-analysis adhered to the recommendations of the Preferred Reporting Items for Systematic Reviews and Meta-Analyses (PRISMA) principles [20]. This study is registered at https://www.crd.york.ac.uk/PROSPERO/; Registration number: CRD42022384532. Potentially eligible articles published up to 1 March 2022 were identified from PubMed, Embase, and the Cochrane Central Register of Controlled Trials (CENTRAL). The following search strategy was used: (seizure OR epilepsy OR partial-onset seizures OR focal epilepsy OR generalized epilepsy OR drug-resistant epilepsy OR refractory epilepsy OR uncontrolled seizure) AND (perampanel OR fycompa OR E2007) in the title/abstract. The subjects of these studies were defined as humans, and the languages of the articles were limited to English. If more than one article had been published using the same data, only the article with the largest number of patients and relatively comprehensive duration of follow-up was considered for the final data analysis. Additionally, a manual search of the reference lists from all identified eligible articles was also conducted.

### Study inclusion criteria

The following inclusion criteria were used in this systematic meta-analysis: 1) recruited patients were ≥12 years of age and had a clinical diagnosis of epilepsy (which included all types of epilepsy), according to the 2017 ILAE classification (Fisher et al., 2017); 2) observational studies (excluding RCTs) with a treatment duration (excluding titration) of ≥3 months because the treatment duration of ≥8 weeks was considered to represent the minimum period for differentiating change in seizure frequency; 3) trial usage of PER as monotherapy or adjunctive treatment; 4) articles written in English with full text available; 5) the provision of at least one of the following outcomes in the studies: 50% responder rate, seizure-free rate, adverse effects (AEs) rate, and withdrawal rate due to AEs; and 6) sample size of ≥10 patients. The following are the exclusion criteria: 1) animal or *in vitro*–based studies; 2) subjects were children and adolescents (aged <12 years); 3) follow-up duration of <3 months; and 4) studies without original data such as commentaries, news items, letters, and reviews.

### Data extraction and outcome measures

All identified articles were independently evaluated for relevance by two authors (XZ and NL) on the basis of the title and abstract; any discrepancies were resolved through discussion with the senior author (QW). Full texts of the selected articles were then checked.

The following information was extracted from each study using a data extraction form (see Table 1): first author and date of publication, trial design (duration of follow-up, dosage of PER, and therapeutic regiment), patients’ demographic information (age, sex of patients, total number of participants, number of concomitant AEDs, and seizure types), and outcomes (efficacy and safety outcomes described as follows).

**TABLE 1:** Characteristics of the observational studies included in this meta-analysis.

First author and year	Number of patients	Study design	Type of concomitant AEDs	Sex (male/female)	Age, years [mean ± SD or mean (range)]	Duration of epilepsy (years/months ± SD)	Duration of follow-up (month)	PER dosage (mg/day) [mean ± SD or mean (range)]	Seizure classification, number of patients			
									A	B	C	D
Alsaadi. T (2019)	21	Add-on	LEV, TPM, VPA, etc.	9/12	27.48 ± 9.72	NA	6	Mean, 7.90	TC, 16	Absence, 2	Atonic, 1	—
Abril. Jaramillo. J (2020)	42	First add-on	CBZ, OXC, LCM, LTG, PHT, etc.	24/18	42.5 ± 5.2	4.7 (0.1–32.2)	6 and 12	6.3	SPS, 16	CPS, 9	FBTCS, 26	
	71	Second add-on	CBZ, OXC, LCM, LTG, PHT, etc.	34/37	38.9 ± 4.1	4.4 (0–31.2)	6 and 12	4–12	SPS, 29	CPS, 49	FBTCS, 33	
Zhang.R. (2021)	56	Add-on	VPA, LEV, CBZ, LTG, CLN, etc.	27/29	30.1 ± 16.3	8.9 ± 8.8	3 and 6	5.0 ± 1.5	POS, 20	sGTCS, 36		
Youn. S.E (2018)	81	Add-on	CBZ, OXC, etc.	44/37	17 (12–32)	NA	3	2–12	POS, 81	FBTCS, 48	UC, 44	
Yamamoto. T (2020)	89	Monotherapy	—	45/44	42.1 ± 18.2	2.1 ± 12.8 months	8	4.8	CPS, 14	SPS, 54	FBTCS, 57	
Wehner. T (2017)	386^a^	Add-on	CBZ, LEV, LTG, etc.	157/219	17–82	NA	6	7.3 ± 3.1	POS, 314	GTCS, 58	UC, 4	
Villanueva. V (2018)	149	Add-on	LEV, VPA, LTG, CLN, etc.	73/76	15 (11–20)	14 (5–29)	3, 6, and 12	5.6	UC, 139	GTCS, 115	MS, 48	Absence, 47
Villanueva. V (2016)	464	Add-on	LEV, CBZ, LTG, etc.	229/235	40.5 (12–82)	24 (14.5–36)	3, 6, and 12	6.3 ± 2.1	SPS, 98	CPS, 363	SGS, 127	—
Vecht. C (2017)	12	Add-on	NA	9/3	41 (31–65)	NA	6	2–12	DRE + SPS, 7	DRE + CPS, 4	DRE + GS, 1	DRE + POS, 2
Usui. N (2018)	30	Study 231, add-on	CBA, TPM, LTG, etc.	14/16	35.4 ± 10.6	NA	6	2–12	SPS, 20	CPS, 51	sGTCS, 22	
	21	Study 233, add-on	VPA, TPM, LTG, etc.	10/11	36.5 ± 11.0	NA	6	2–12	SPS, 12	CPS, 34	sGTCS, 14	
Toledano. D.R (2020)	98	Monotherapy	None	48/50	49.6 ± 21.7	6.5 (2–13)	3, 6, and 12	4 (2–10)	SPS, 14	CPS, 18	GTCS, 33	
Takahashi. S (2019)	8	First add-on	LEV, VPA, LTG, etc.	2/6	49.1 ± 14.7	NA	6	3 (2–8)	POS, 8			
	22	Late add-on	LEV, VPA, LTG, etc.	15/7	35.0 ± 14.8	NA	6	4 (2–8)	POS, 22			
	5	First add-on	LEV, VPA, LTG, etc.	2/3	44.8 ± 12.0	NA	12	2 (2–8)	POS, 5			
	16	Late add-on	LEV, VPA, LTG, etc.	11/5	37.0 ± 14.5	NA	12	4.4 (2–8)	POS, 16			
Steinhoff. B.J (2014)	74	Add-on	EIAEDs and non-EIAEDs	31/43	38.4 (15–71)	NA	6	8.8 (4–14)	POS, 71	LGS, 4		
Stavropoulos.I (2019)	181	Add-on	SCB, non-SCB, and mixture	88/93	41.2 ± 12.8	27.5 ± 13.7	12	2–12	POS, 134	GTCS, 44	UC, 3	
Shankar. R (2017)	144	Add-on	NA	72/71	44 (20–76)	NA	3, 6, and 12	2–12	ID + POS, 73	ID + GTCS, 68	ID + FBTCS, 3	
Shah. E (2016)	310	Add-on	NA	155/155	18–75	26.7 ± 13.5	6	7.1 ± 2.9	POS, 230	IGE, 8	SGE, 15	UC, 57
Estevo Santamarina.E (2020)	149	Add-on	LTG, VPA, ESL, CBZ, etc.	81/68	41 (12–84)	9.6 ± 11.4	12	6.2	POS, 113	GS, 32		
Sagar. P (2021)	387	Add-on	CBZ, OXC, LCM, LTG, PHT, etc.	175/212	≥16	21 ± 31.2	3, 6, and 12	8 (4–8)	DRE + POS, 309	DRE + IGE, 40	DRE + DEE, 38	
Rodríguez-Osorio.X (2021)	77	Add-on	LEV, CBZ, PHT, VPA, etc.	45/32	46 (33–58.5)	NA	3, 6, and 12	4 (4–8)	FIAS, 46	FBTCS, 25	Other POS, 12	
Rinaldi. F (2018)	52	Add-on	CBZ, OXC, PHT, etc.	18/34	38.7 ± 12.4	28.1 ± 12.6	12	7.57 ± 2.5	DRE + POS, 38	DRE + FBTCS, 11		
Rektor. I (2012)	138	Add-on	CBZ, LTG, PHT, etc.	58/80	40.7 ± 11.9	23.2 ± 13.4	12	2–12	SPS, 64	CPS, 131	FBTCS, 88	
Pascarella. A (2020)	246	Add-on	CBZ, OXC, PHT, etc.	111/135	37.9 ± 13.7	24.3 ± 13.5	6 and 12	6.5 ± 2.1	POS, 124	sGTCS, 122		
Nilo. A (2021)	63	Add-on	EIAEDs	31/32	45.8 ± 12.8	26 (1–60)	12	5.9 ± 1.93	POS, 32	FBTCS, 6	POS + FBTCS, 25	
Moraes. J.S (2020)	160	Add-on	SV2A inhibitor, SCB, GABA analog, and MM AD	78/82	40.4 ± 13.3	21.7 ± 14.8	6 and 12	4–12	POS, 160			
Maschio. M(2020)	26	Add-on	LCM, LEV, VPA, and LTG	16/10	47.5	NA	6	2–12	Brain tumor-related epilepsy, 26			
Maschio. M(2019)	11	Add-on	LEV, LTG, CBZ, LCM, ZNS, and VPA	9/2	Mean 54	NA	12	7.3 ± 1.6	POS, 5	FBTCS, 6		
Lossius. I.M.B (2021)	175	Add-on	LTG, VPA, and LEV	81/94	32 (3–75)	12 (0–66)	12	6.3 ± 3	POS, 140	GS, 25	UC, 10	
Lin. C.Y (2019)	44	Add-on	LEV, OXC, VPA, and LTG	20/24	42.0 ± 13.3	21.9 ± 11.9	6	5.56 (2–12)	SPS, 1	CPS, 9	FBTCS, 34	
Limotai. C (2021)	35	Add-on	CBZ, LEV, LTG, etc.	13/22	40.06 ± 12.34	23 ± 8.51	3 and 12	2–8	DRE, 35			
Liguori. C (2020)	64	Add-on	VPA, LEV, CBZ, LCM, TPM, etc.	37/27	43 ± 17.44	18.78 ± 14.05	12	4.8 ± 1.79	POS, 45	GS, 12	FBTCS, 7	
Liguori. C (2018)	15	Add-on	CBZ, VPA, OXC, ZNS, etc.	8/7	40 ± 18.53	15.13 ± 10.33	3, 6, and 12	5.42 ± 2.51	sGS, 15			
Lattanzi. S (2021)	92	Add-on	EIAEDs and non-EIAEDs	46/46	69 (66–73)	22 (7–49)	3, 6, and 12	6 (4–6)	POS, 73	GS, 5	FBTCS, 22	
Labate. A (2021)	20	First add-on	LEV, LTG, CBZ, LAC, etc.	6/14	43.55 ± 14.68	17.80 ± 12.7	3 and 12	4.2 ± 1.28	MTLE, 20			
	17	Second add-on	LEV, LTG, CBZ, LAC, etc.	4/13	48.06 ± 14.87	23.±10.37	3 and 12	5.8 ± 2.17	MTLE, 17			
Kurth. C (2017)	70	Add-on	TPM, VPA, LEV, CBZ, OXC, etc.	29/41	38.9 (15–71)	24.0 (7–56)	6	8.6 (4–14)	DRE, 70			
Krauss. G.L (2018)	1,218	Add-on	NA	448/446	30–34	19.6 (0–33)	12, 24, 36, and 48	10.1 ± 2.3	POS, 1,218			
Krauss. G.L (2014)	1,216	Add-on	NA	610/606	34.3 (12–76)	19.6 (0–33)	12	10.6 ± 2.25	POS, 1,218			
Kim. S.Y (2018)	97	Add-on	NA	62/35	5.2 (0–15.4)	15.7 (4.3–25.3)	12	6.6 (2–12)	POS, 97			
Kim. J.H (2020)	85	Add-on	SCB, SV2A Antagonism, and multiple mechanisms	36/49	42.3 ± 14.1	10.9 ± 9.3	6	2–12	POS, 85	SG, 16		
Kim. D.W (2017)	137	Add-on	NA	86/51	38.9 ± 14.4	NA	6	4.39 ± 1.97	POS, 118	GS, 19		
Kanemura.H (2019)	41^a^	Add-on	LEV, CBZ, VPA, etc.	21/18	13.6 (12–18)	8.61 (5.3–12.3)	12	6.94 (4–12)	POS, 13	UC, 3	FBTCS, 29	
Juhl. S (2016)	22	Add-on	LEV, LTG, LAC, etc.	10/12	36.4 (20–64)	21 (3–55)	12	5.8 (4–10)	SPS, 7	CPS, 22	sGS, 16	
Inoue. Y (2022)	3,716	Add-on	NA	1965/1751	45.0 ± 19.0	NA	12	3.7 ± 1.9	FS with or without FBTCS, 2,517	GTCS, 426	UC, 117	
Ikemoto. S (2019)	84^a^	Add-on	LEV, CBZ, OXC, etc.	33/41	43.3 ± 14.2	NA	12, 24, and 36	4–10	POS, 74			
Husni. R.E (2021)	89	Monotherapy	None	34/55	12–74	0 (0–10)	12	4	SPS, 13	CPS, 41	FBTCS, 48	
Huber. B (2017)	26	Add-on	LTG, VPA, OXC, RTG, etc.	11/15	30 (21–55)	NA	6 and 12	8 (4–10)	DRE + ID, 26			
Gil-Nagel. A (2018)	60	Monotherapy	—	22/38	≥12	Most ≥1 year	3, 6, and 12	2–12	POS, 60			
Gil-López. F.J (2018)	31	Add-on	LEV, VPA, and ZNS	12/19	36.4 ± 14.1	18 (8–26)	3 and 6	6	MS, 31	GTCS, 17	Absence, 5	GTS, 4; LRE, 1
Garamendi-Ruiz I (2016)	256	Add-on	LEV, CBZ, ESL, TPM, etc.	113/143	39.1 ± 12.75	NA	6 and 12	2–12	POS, 157	FBTCS, 89	GS, 10	
Davis. Jones. G (2021)	113	Add-on	EIAEDs and sodium valproate	53/60	≥18	NA	6	6.8 ± 3.3	Post-VNS, 77	Post-resective surgery, 36		
Coppola. A (2020)	36	Add-on	LEV, PB, OXC, etc.	23/13	46 (15–75)	NA	12	2–12	SPS, 14	CPS, 7	FBTCS, 11	GS, 4
Chiang. H.I (2017)	210^a^	Add-on	EIAEDs and non-EIAEDs	86/73	38.1 ± 12.6	20.1 ± 12.0	3	5.31 (2–12)	LRE, 17	FBTCS, 130	GS, 12	
Canas. N (2021)	21	First add-on	NA	10/11	35 (20–59)	5.0 (2.5–9.5)	3, 6, and 12	4	SPS, 0	CPS, 6	FBTCS, 16	
	60	Late add-on	NA	31/29	43 (30–55)	26 (16.0–36.0)	3, 6, and 12	6	SPS, 12	CPS, 40	FBTCS, 18	
Brodie. M.J (2016)	54	Add-on	LEV, CBZ, VPA, LTG, etc.	38/16	48 (21–65)	4 (1–60)	6	4 (4–12)	POS, 54			
Basheikh. M(2020)	102	Add-on	NA	47/55	40.25 (18–72)	0.77 (0.5–2.5)	6	2–12	LRE, 87	GS, 13	UC, 2	
Chinvarun. Y (2022)	41	Monotherapy	None	17/24	46.1 ± 21.8	3.57 (0–5)	3, 6, and 12	4 (2–8)	POS, 41			
Zhang. Y (2022)	72	Add-on	VPA, LEV, OXC, LTG, etc.	41/31	27.28 ± 12.86	12.44 ± 9.79	6	4.96 ± 2.41 (2–12)	LRE, 69	GS, 3		

Note: AEDs, antiepileptic drugs; SD, standard deviation; TC, tonic–clonic; NS, the nocturnal seizure group; NA, not available; SPS, simple partial seizure; CPS, complex partial seizures; FBTCS, focal to bilateral tonic–clonic seizures; POS, partial-onset seizures; sGTCS, secondarily generalized tonic–clonic seizures; UC, unclassified; GTCS, generalized tonic–clonic seizures; MS, myoclonic seizure; SGS, secondarily generalized seizures; DRE, drug-resistant epilepsy; GS, generalized seizures; ID, intellectual disability; IGE, idiopathic generalized epilepsy; SGE, symptomatic generalized epilepsy; GS, generalized seizures; NS, nocturnal seizure; DEE, epileptic encephalopathy; FIAS, focal impaired awareness seizure; sGS, focal and generalized seizures; MTLE, mesial temporal lobe epilepsy; LRE, localization-related epilepsy (focal epilepsy); post-VNS, people with epilepsy undergone vagus nerve implantation; post-resection, people with epilepsy undergone surgical resection.

Abbreviation of concomitant drugs: LEV, levetiracetam; TPM, topiramate; VPA, valproic acid; CBZ, carbamazepine; OXC, oxcarbazepine; LCM, lacosamide; LTG, lamotrigine; PHT, phenytoin; CLN, chlordiazepoxide; ZNS, zonisamide; RTG, retigabine; EIAEDs, enzyme-inducing AEDs; SCB, drugs acting on sodium channels; MM AD, multiple mechanisms; SV2A, synaptic vesicle glycoprotein 2A.

^a^ Baseline characteristics for patients were based on the modified intent-to-treat (mITT) population set.

The number of participants experiencing any seizure-related outcome and the total number of participants were extracted. In this meta-analysis, the primary efficacy outcomes analyzed were the 50% responder rate (proportion of patients with ≥50% reduction in seizure frequency in the treatment period when compared with the pretreatment baseline period) and seizure-free rate (proportion of patients who were seizure free during treatment and the follow-up period). The secondary efficacy outcomes analyzed were retention rates (proportion of patients who continued treatment at the end of the follow-up period), AEs (proportion of patients who experienced at least one of the common AEs after receiving at least one dose of PER), and withdrawal rate due to AEs (proportion of patients who experienced at least one AE with PER treatment and withdrawal during the course of the treatment period) were analyzed to evaluate the safety of PER usage.

It is worth noting that across these observational studies, the seizure outcomes were defined in different populations. Some studies were based on the modified intent-to-treat (mITT) population set (the mITT analysis set included all patients who received at least one dose of the study drug and had any seizure frequency data collected during the PER treatment duration), while some studies reported in the completer population. Due to variations in the denominators assessing these seizure outcomes used across the studies, all analyses in this meta-analysis were conducted in the ‘full analysis set’ (which included all individuals who took at least one dose of PER). We recalculated the N-numbers of each outcome data according to the ratio and corresponding denominators/populations if these specific N-numbers of events were not given in the original studies.

### Data synthesis and analysis

Data analysis was performed using STATA version 16.0 (StataCorp LP, College Station, TX). The degree of between-study heterogeneity was analyzed using the Cochran’s Q and I^2^ tests, with I^2^ ≥ 40% or *p* ≤ 0.1 for the Q test, indicating significant heterogeneity. When the between-study heterogeneity was identified significantly, data were analyzed using a random-effects model to calculate the pooled rates (PRs) with their corresponding 95% confidence intervals (CIs). Otherwise, a fixed-effects model was used to analyze the data. The potential publication bias was analyzed using Begg’s and Egger’s tests, and a value of *p* < 0.05 was considered statistically significant.

Considering that the efficacy and safety of PER usage could be affected by various factors, the following subgroup analyses (50% responder rate, seizure freedom, AE rate, and retention rate) were performed. Comparisons between the subgroups were based on dose titration (slow titration *vs*. fast titration), treatment regimen (primary monotherapy *vs*. conversion monotherapy), interactions of concomitant AEDs (with *vs*. without EIAEDs), study duration (short-term follow-up of 3 months and 6 months *vs.* long-term follow-up ≥12 months), and PER add-on therapeutic schedule (first *vs*. late add-on therapy) were performed.

Open-label extension (OLEx) Study 207 (patients were required to have completed phase IIa dose-finding Study 206 or 208) and Study 307 (patients were required to have phase III dose-finding Study 304, 305, or 306) examined the long-term efficacy and safety of high-dose PER usage as an adjunctive therapy in patients with refractory partial-onset seizures. However, in Studies 207 and 307, the dosage and types of AEDs could be adjusted, changed, or discontinued during the OLEx period. Considering long-term extension studies have different designs from those of observational studies, the data were analyzed separately.

## Results

### Characteristics of included studies

**FIGURE 1:**
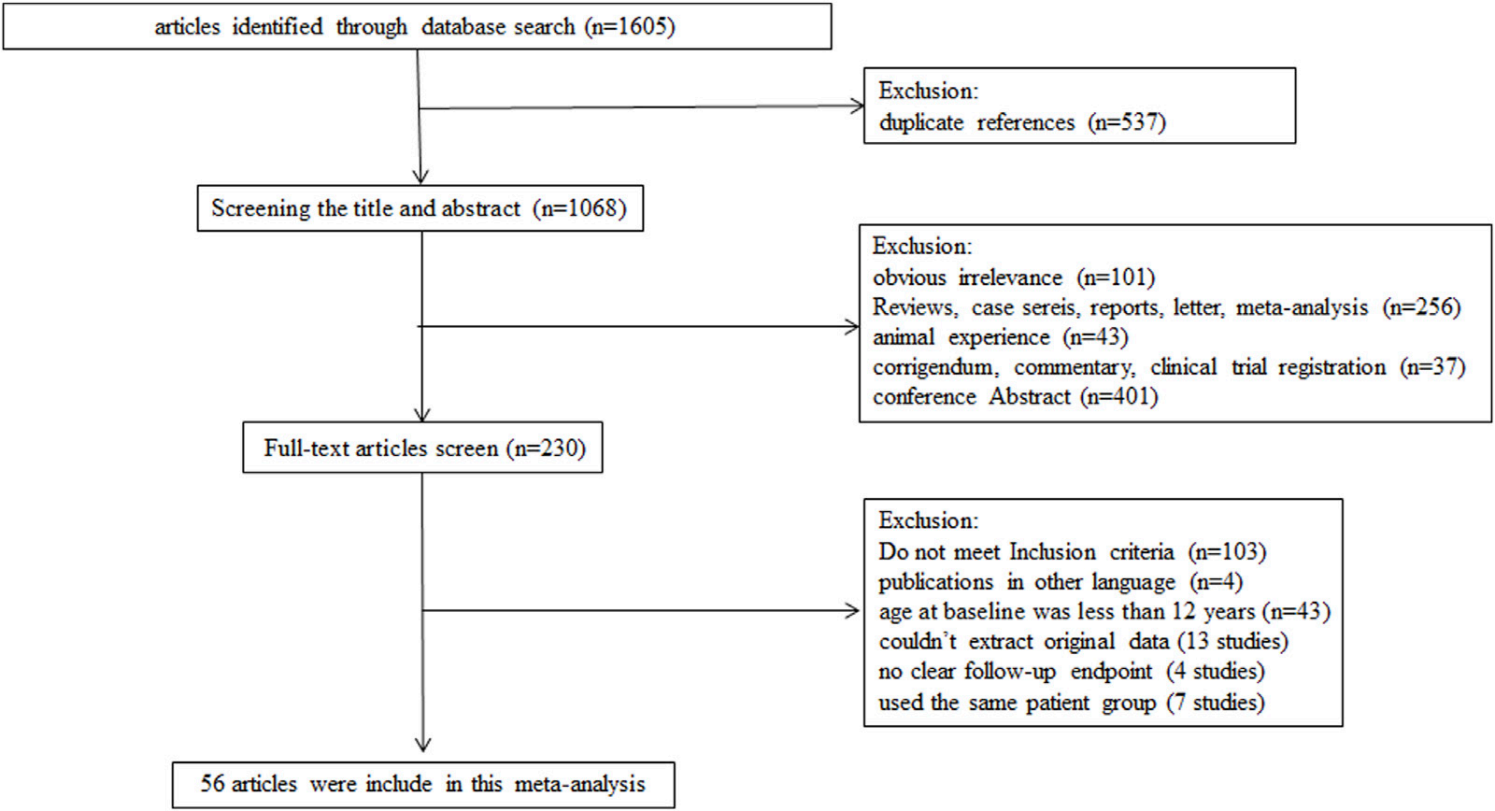
Flow diagram of study process.

The electronic search in the PubMed, Embase, and Cochrane Library databases identified a total of 1,605 potentially relevant articles. After removing the duplicate studies, 1,068 studies were independently screened for title and abstract. Among these, 838 studies were excluded because of obvious irrelevance, animal studies, reviews, commentaries, case reports, letters, and conference abstracts. After detailed assessment of the remaining 230 full-text articles, 174 studies were further excluded because they did not meet the inclusion criteria (103 studies), were published in other languages without other available details (4 studies), included subjects who were children <12 years (43 studies), had no clear follow-up endpoint (4 studies), and had duplicate reports with no additional data (7 studies), as well as those whose original data could not be extracted (13 studies). Finally, 56 studies met the inclusion criteria and were enrolled (Rektor et al., 2012; Krauss et al., 2014; Steinhoff et al., 2014; Brodie and Stephen, 2016; Garamendi-Ruiz et al., 2016; Juhl and Rubboli, 2016; Shah et al., 2016; Villanueva et al., 2016; Chiang et al., 2017; Huber and Schmid, 2017; Kurth et al., 2017; Shankar et al., 2017; Vecht et al., 2017; Wehner et al., 2017; Gil-López et al., 2018; Gil-Nagel et al., 2018; Kim et al., 2018; Kim and Oh, 2018; Krauss et al., 2018; Liguori et al., 2018; Rinaldi and De Maria, 2018; Usui et al., 2018; Villanueva et al., 2018; Youn et al., 2018; Alsaadi et al., 2019; Ikemoto et al., 2019; Kanemura et al., 2019; Lin et al., 2019; Maschio et al., 2019; Stavropoulos et al., 2019; Takahashi et al., 2019; Abril Jaramillo et al., 2020; Coppola et al., 2020; Kim et al., 2020; Liguori et al., 2020; Maschio et al., 2020; Moraes et al., 2020; Pascarella et al., 2020; Santamarina et al., 2020; Toledano Delgado et al., 2020; Yamamoto et al., 2020; Basheikh and Sadler, 2021; Canas et al., 2021; Davis Jones et al., 2021; Im et al., 2021; Inoue et al., 2021; Labate et al., 2021; Lattanzi et al., 2021; Limotai and Jirasakuldej, 2021; Lossius et al., 2021; Nilo et al., 2021; Rodríguez-Osorio et al., 2021; Sagar et al., 2021; Zhang et al., 2021; Chinvarun, 2022; Husni et al., 2022). A diagram summarizing the process of study selection is shown in Figure 1.

### Characteristics of included studies

A total of 56 studies, which included 10,688 patients, were enrolled in this meta-analysis. The main characteristics of the included studies are presented in Table 1. All studies were published from 2012 to 2022. The sample size in these studies ranged from 11 to 3,716. The median duration of follow-up ranged from 3 months to 4 years, whereas only two studies (OLE Study 207 and 307) were performed for more than 1 year. The outcomes in most studies were assessed at 3, 6, and 12 months of PER treatment in comparison to the baseline. Usually, at least 12 months of follow-up was required to draw a conclusion on the long-term efficacy and safety of AED treatment, and 32 studies fulfilled this requirement.

### Clinical efficacy outcomes

**FIGURE 2:**
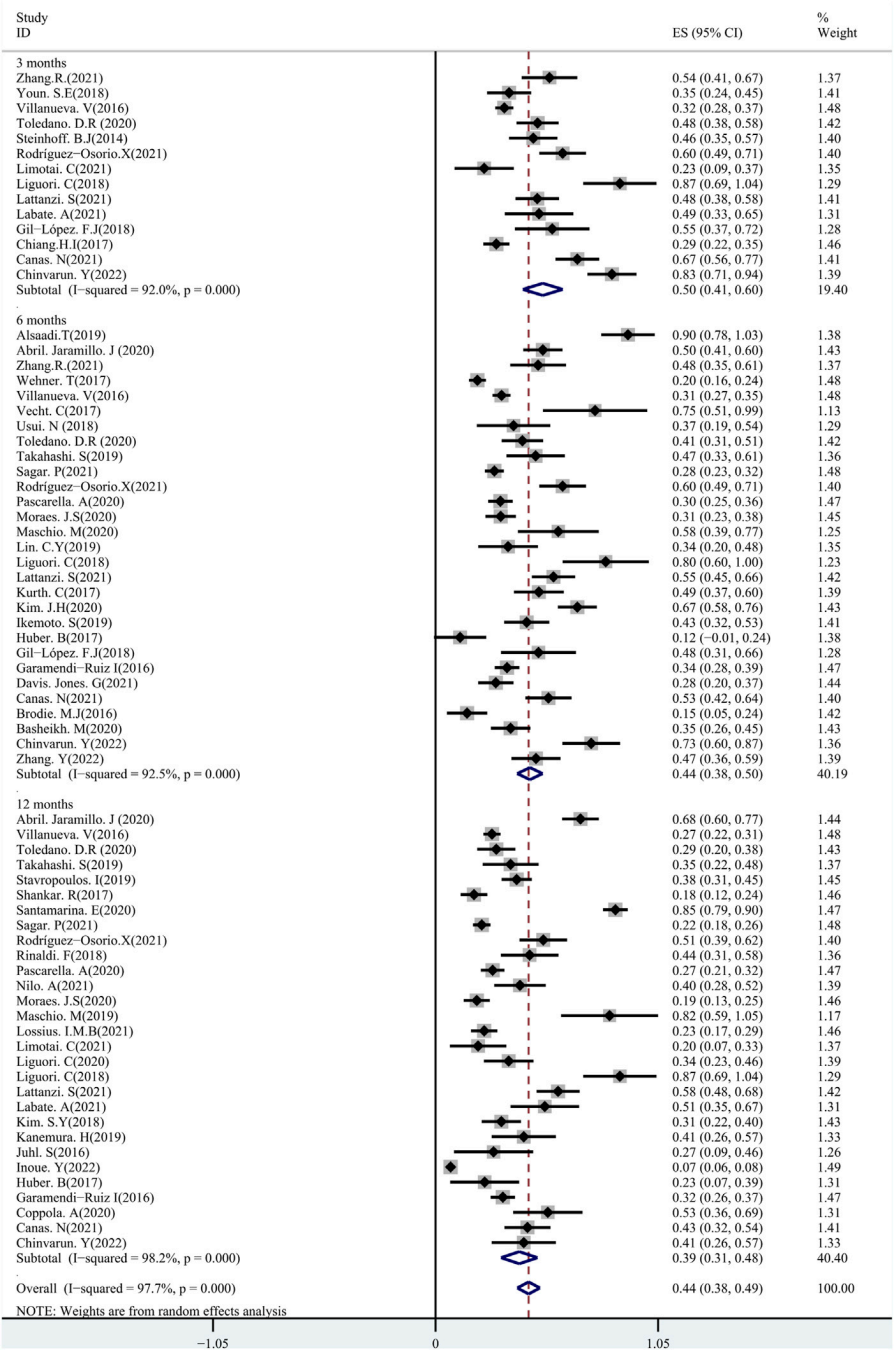
Meta-analysis of 50% responder rate: pooled data from 48 studies.

**FIGURE 3:**
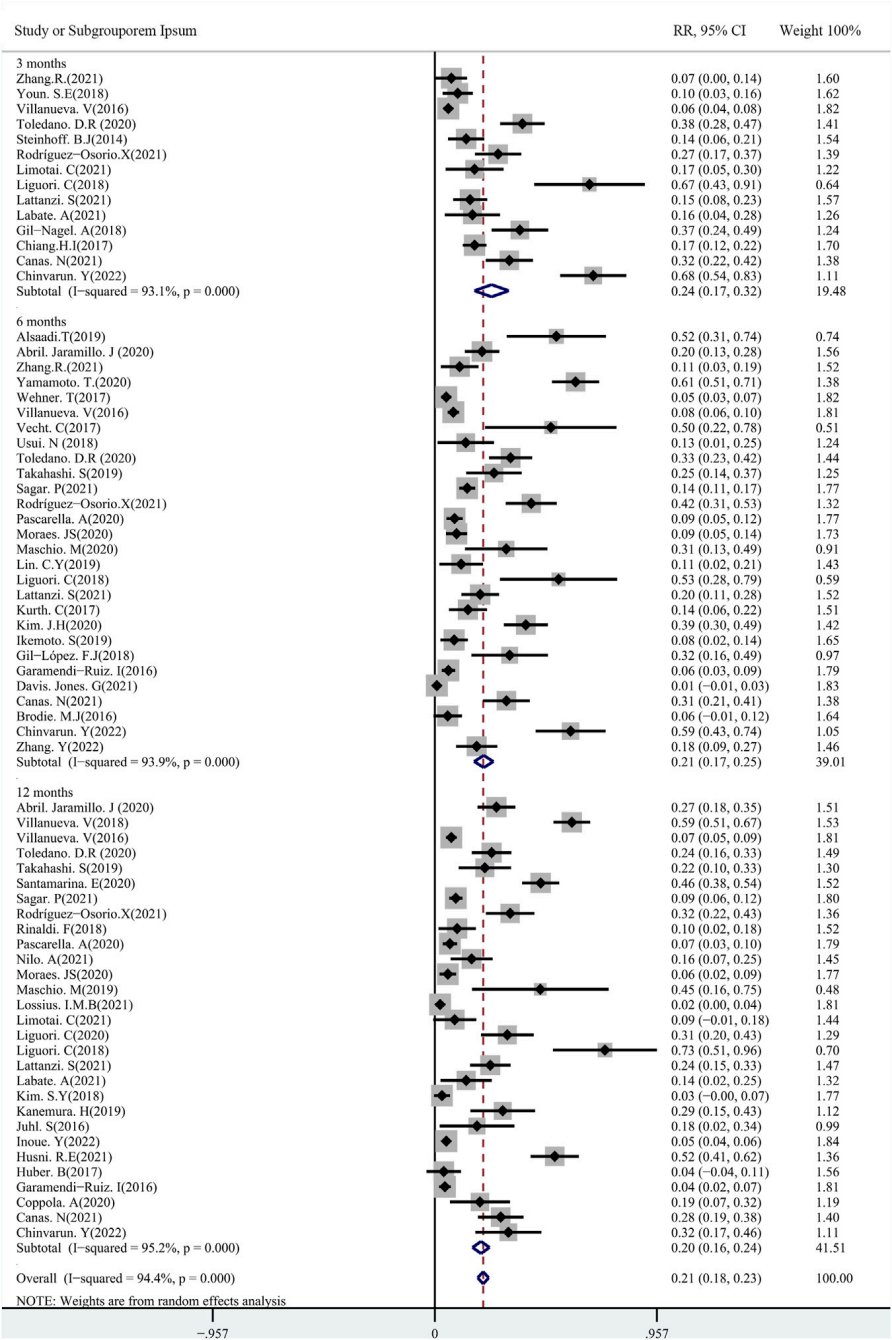
Meta-analysis of seizure-free rate: pooled data from 47 studies.

A total of 48 studies provided data regarding seizure-frequency reduction from the baseline ≥50%, in which 19 studies were carried out at different time points. We, therefore, analyzed the data separately according to the length of follow-up (3, 6, and 12 months after PER treatment) and considered them to be separate data. The 50% responder rates of PER treatment ranging from 7.08% to 90.5% were available for analysis in 8,524 patients. Due to important heterogeneity (heterogeneity: *p* = 0.00, I^2^ = 97.7%), a random-effects model was used to calculate the pooled relative risk (RR) and corresponding 95% CIs. The pooled 50% responder rates were 50.0% (95% CI: 0.41–0.60) and 44.0% (95% CI: 0.38–0.50) after 3 and 6 months of PER treatment, respectively (Figure 2). Furthermore, the 50% responder rates for long-term (12-month follow-up) PER treatment were analyzed from the data provided by the 4,116 patients in the 29 studies, and a range of 7.08%–86.7% for the 50% responder rates was observed. The pooled 50% responder rate for long-term PER treatment was 39.0% (95% CI: 0.31–0.48) (Figure 2). We performed sensitivity analysis by excluding one study that had the maximum sample size with the least effectiveness (Inoue et al., 2021), and the pooled 50% responder rate for long-term PER treatment changed from 39.0% (95% CI: 0.31–0.48) to 40.4% (95% CI: 0.33–0.48). No publication bias was seen based on the Begg’s analysis (*p* = 0.06). The data regarding seizure-free rates (ranging from 3.0% to 73.0%) were provided in 47 studies (including 8,414 patients). The pooled seizure-free rates were 24.0% (95% CI: 0.17–0.32) and 21.0% (95% CI: 0.17–0.25) after 3 and 6 months, respectively, of PER treatment with high heterogeneity (Figure 3). Notably, 29 studies provided seizure freedom outcomes in response to long-term PER treatment. The pooled seizure-free rate at 12 months of follow-up was 20.0% (95% CI: 0.16–0.24) with high heterogeneity (Figure 3). No publication bias was found (*p* = 0.08). Sensitivity analysis, which excluded one study that had the maximum sample size with the least effectiveness, showed that the seizure-free rate of long-term PER treatment changed from 20.0% (95% CI: 0.16–0.24) to 20.1% (95% CI: 0.19–0.24).

Forty-eight studies reported data on the proportion of patients who continued treatment at the end of the follow-up period. In addition, 32 studies provided long-term retention data. The pooled retention rate was 84.0% (95% CI: 0.76–0.91), 74.0% (95% CI: 0.68–0.80), and 69.0% (95% CI: 0.63–0.75) at 3, 6, and 12 months of PER treatment, respectively (Supplementary Figure S1).

### Impact of first or second add-ons on efficacy of PER

**FIGURE 4:**
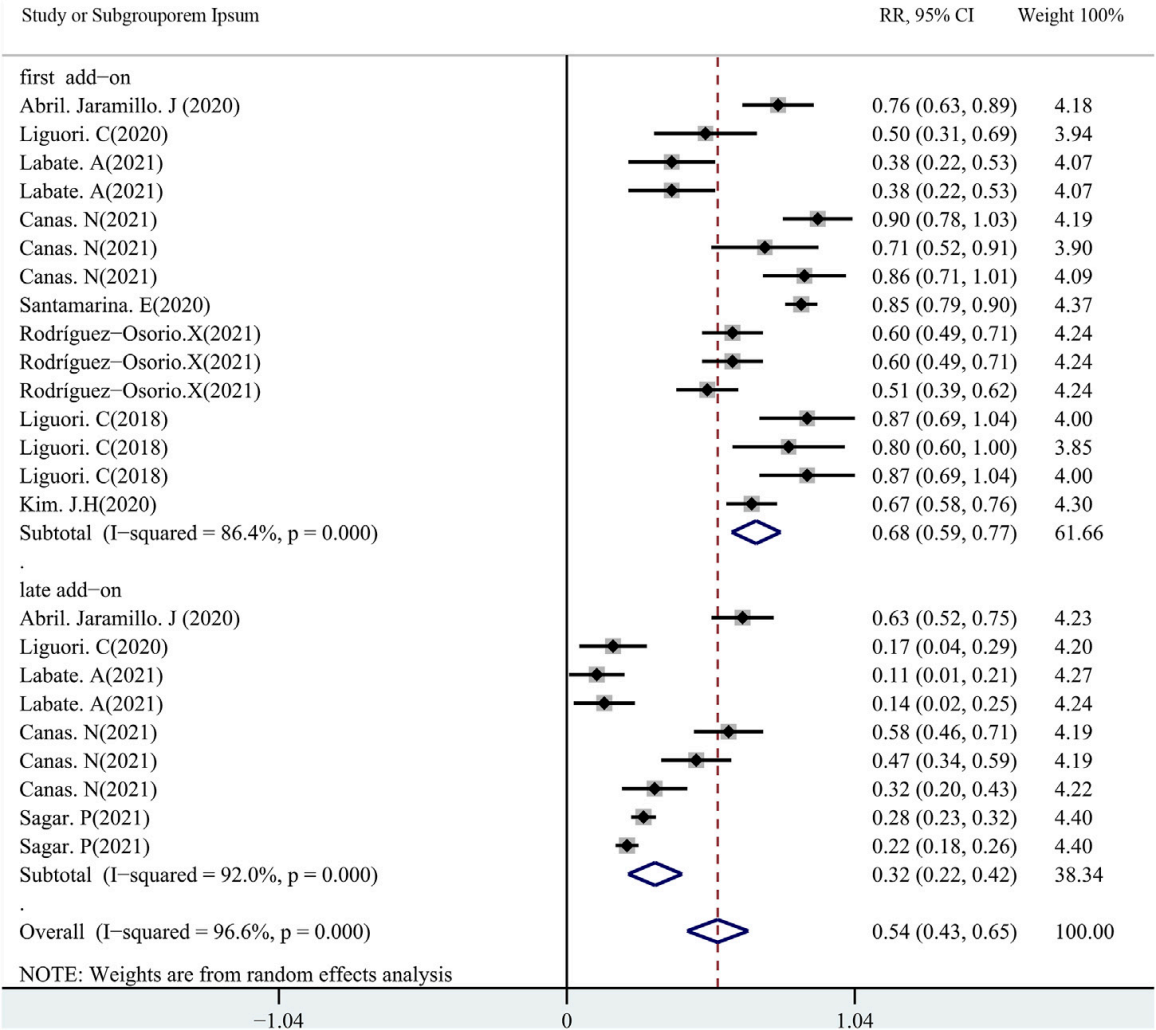
50% responder rate (first *vs*. second add-on).

**FIGURE 5:**
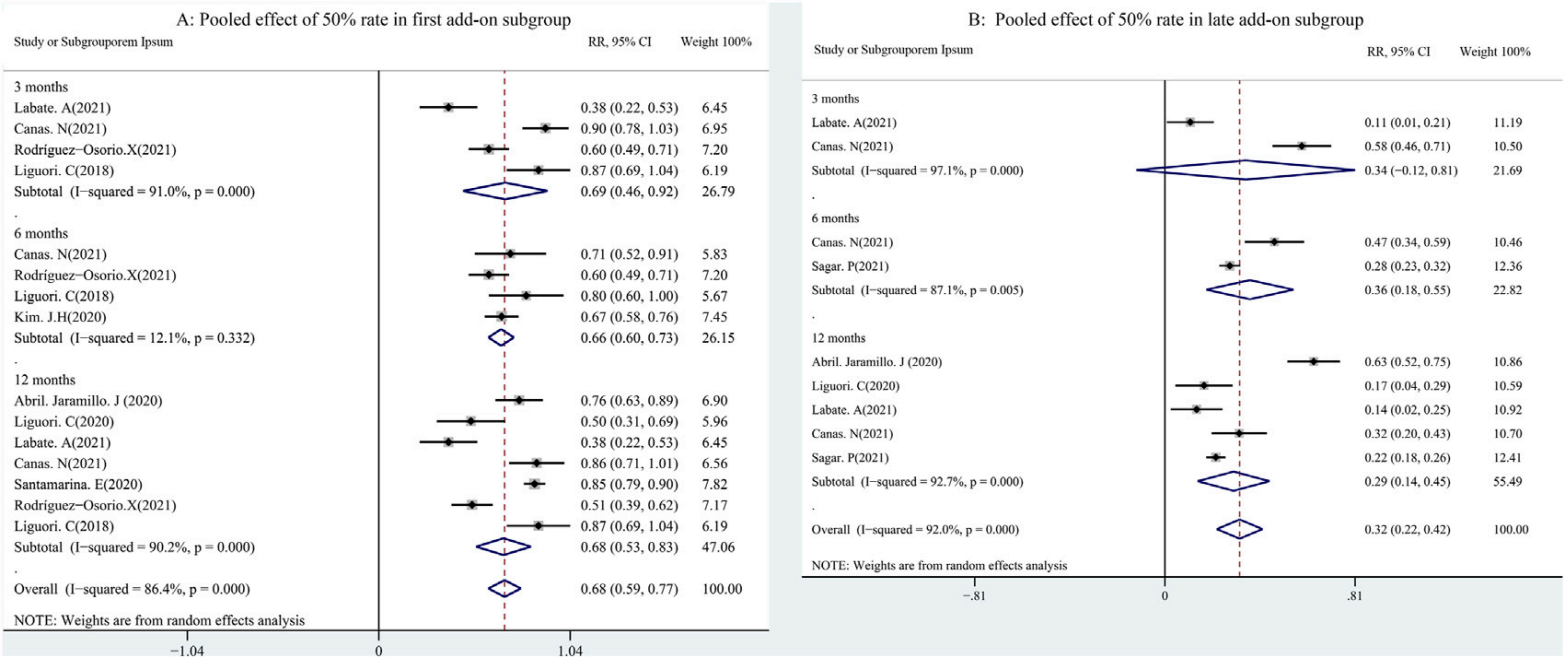
First **(A)** *vs*. late add-on **(B)**: pooled 50% responder rate by follow-up points (3, 6, and 12 months after PER treatment).

**FIGURE 6:**
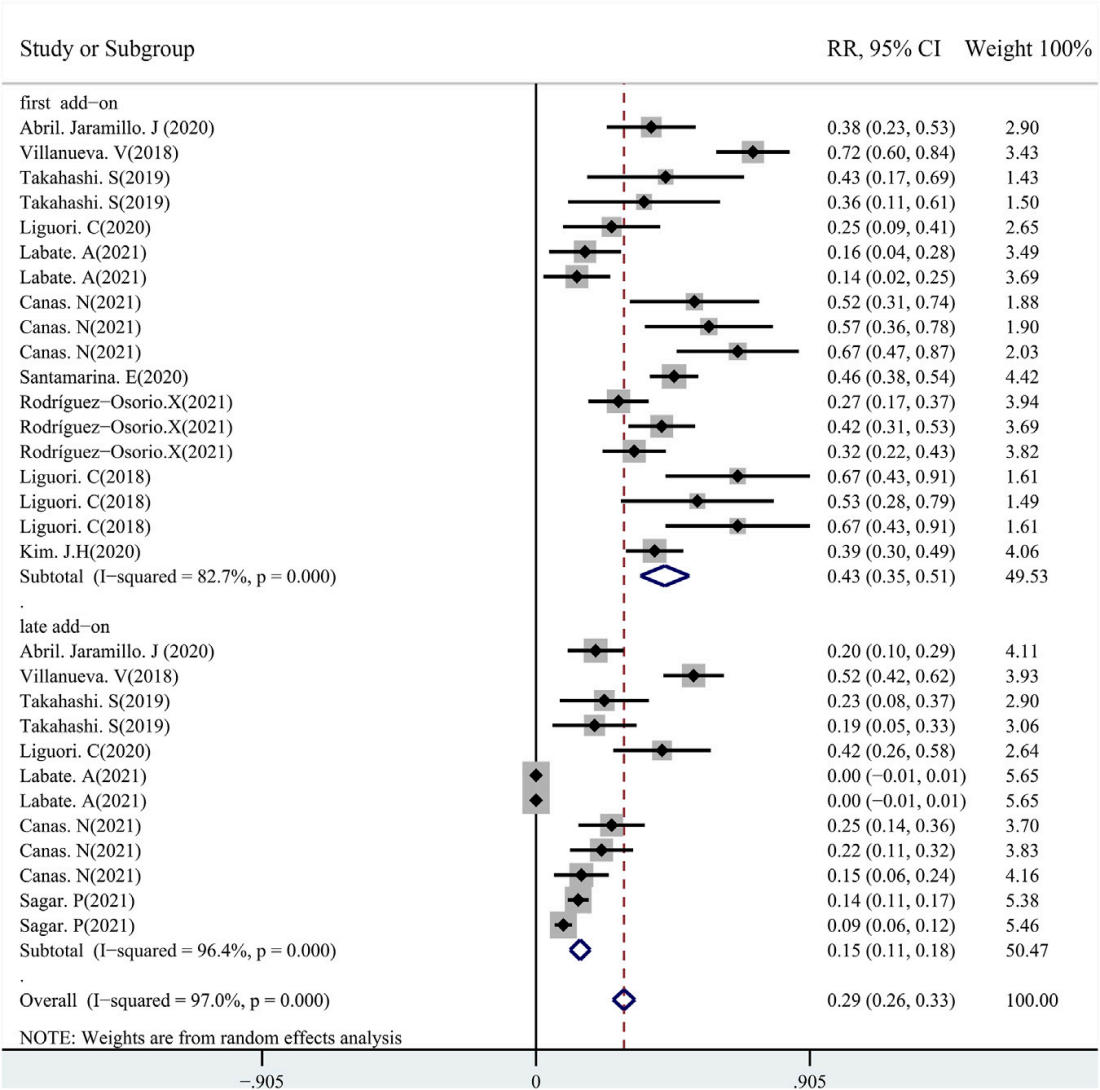
Pooled seizure-free rate (first *vs*. second add-on).

**FIGURE 7:**
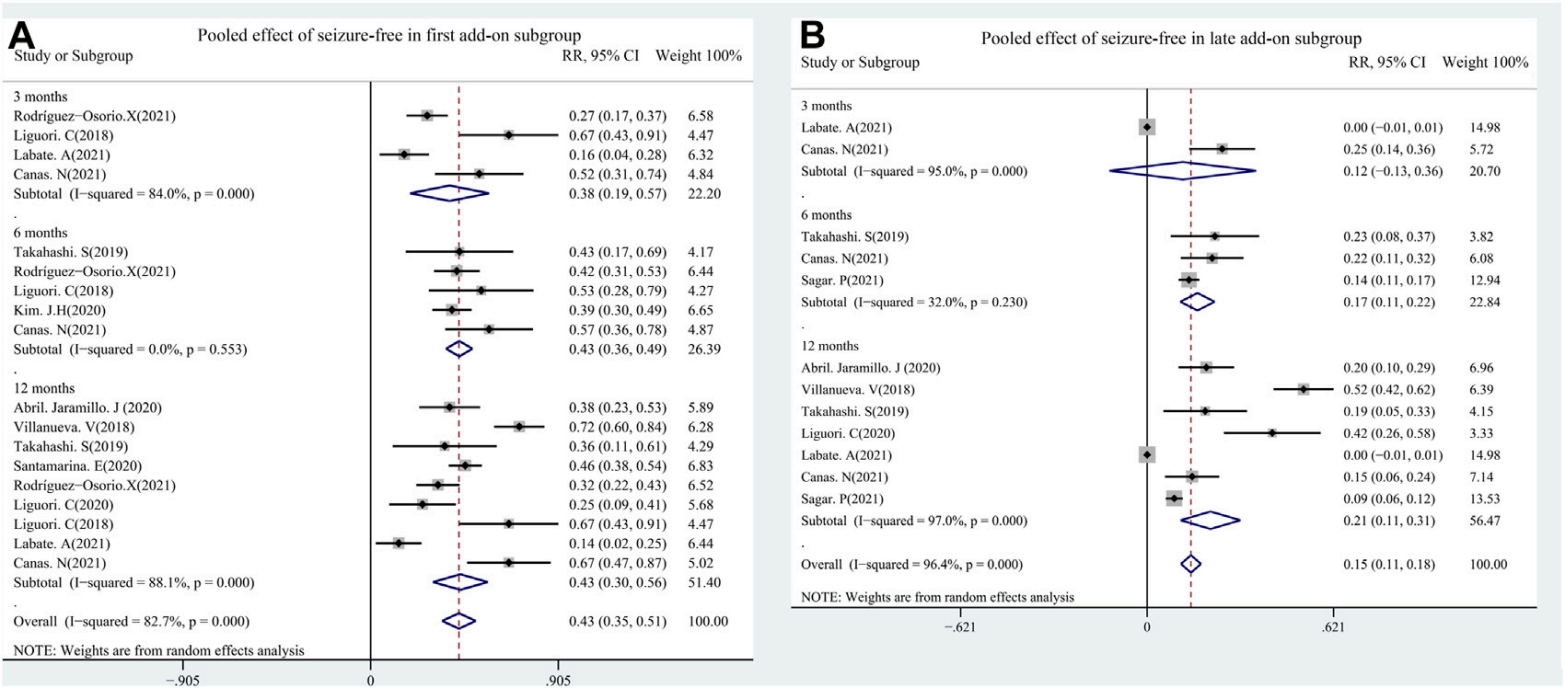
First **(A)** *vs*. late add-on **(B)**: seizure-free rate by follow-up points (3, 6, and 12 months after PER treatment.

A total of 11 studies (including 1,256 patients) were used to assess the effects of the first (early) and/or second (late) add-ons on the efficacy of PER usage in patients with epilepsy. Among the 11 studies, 6 studies compared the efficacy of PER as the first and late add-on treatments, whereas a single first or late add-on treatment was employed in the other 5 studies. The pooled 50% responder rate (including all follow-up points) was 68.0% (95% CI: 0.59–0.77) and 32% (95% CI: 0.22–0.42) in first add-on and late add-on groups, respectively (Figure 4). Due to the variations in the duration of PER treatments in the different studies, we conducted further analysis on the basis of the length of follow-up (3, 6, and 12 months after PER treatment). The first add-on group had a higher pooled 50% responder rate than the late add-on group at 3 months (first add-on group 69%, 95% CI: 0.46–0.92; late add-on: 34%, 95% CI: −0.12–0.81), 6 months (first add-on group 66%, 95% CI: 0.60–0.73; late add-on: 36%, 95% CI: 0.18–0.55), and 12 months (first add-on group 68%, 95% CI: 0.53–0.83; late add-on: 29%, 95% CI: 0.14–0.45) (Figure 5). Similar to that of the 50% responder rate, patients in the first add-on group (43%, 95% CI: 0.35–0.51) displayed higher seizure-free rates than those in the late add-on group (15%, 95% CI: 0.11–0.18) (Figure 6) at different time points after PER treatment (3 months: first add-on group 38%, 95% CI: 0.19–0.57 *vs*. late add-on 12%, 95% CI: −0.13–0.36; 6 months: first add-on group 43%, 95% CI: 0.36–0.49 *vs*. late add-on 17%, 95% CI: 0.11–0.22; and 12 months: first add-on group 43%, 95% CI: 0.30–0.56 *vs*. late add-on 21%, 95% CI: 0.11–0.31) (Figure 7).

### Impact of monotherapy on efficacy of PER

**FIGURE 8:**
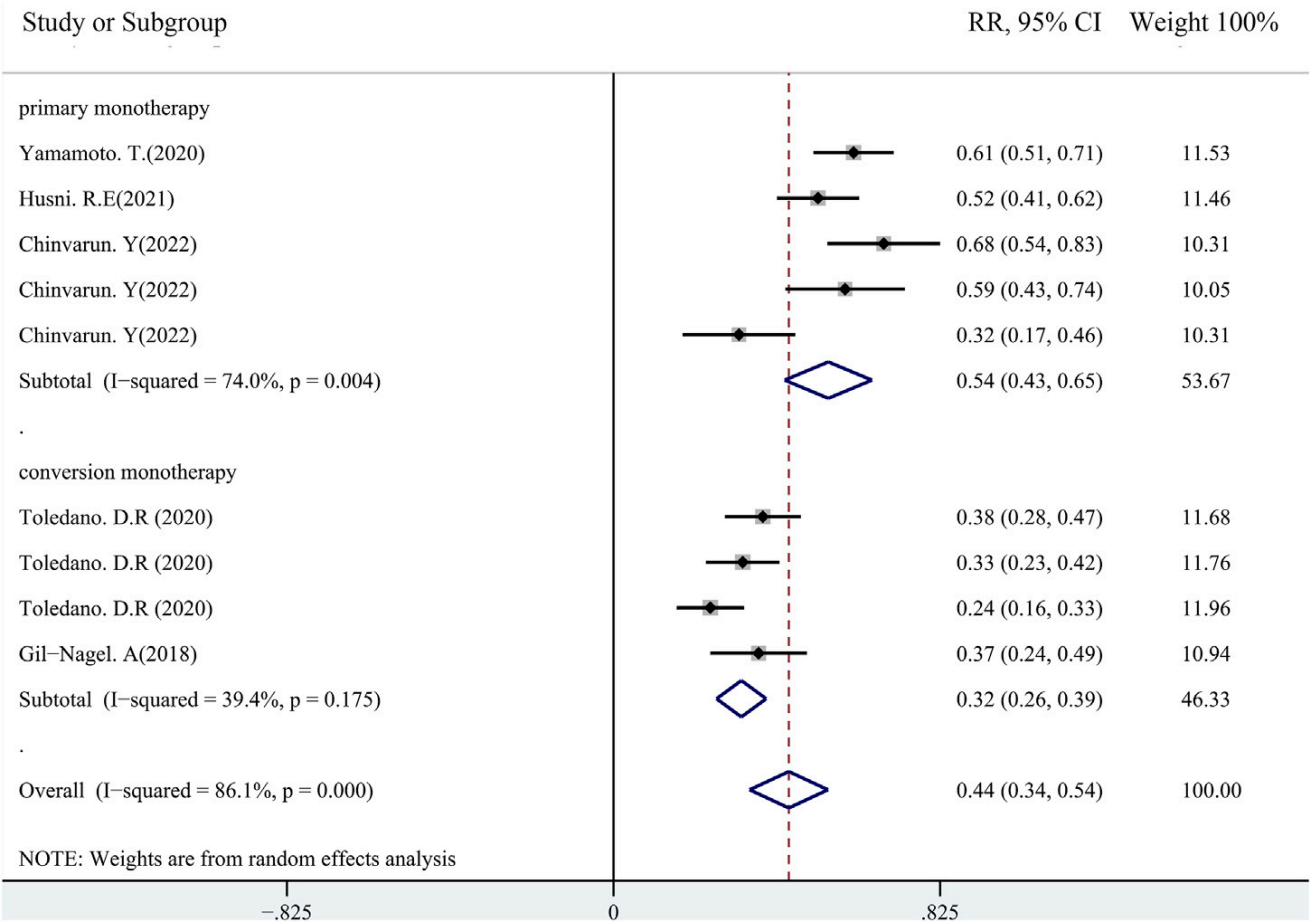
Pooled seizure-free rate (primary *vs*. conversion monotherapy group).

Until now, PER has been prescribed as monotherapy for focal epileptic seizures in the US and Japan only. PER monotherapy includes primary monotherapy (patients were treated with PER only in the absence of any concomitant other AEDs) and conversion monotherapy (patients were treated with one or more AEDs, including PER, until they were included in the study, and they were then converted to PER only). The median PER maintenance dose was 6 mg during monotherapy, although 4 mg was the most common. A total of five studies that were performed from 2018 to 2022 were used to evaluate the impact of monotherapy on the efficacy of PER usage in patients with FOS, in which two studies had been conducted in patients receiving PER as conversion monotherapy and three studies had employed PER as primary monotherapy. The combined outcomes (including all follow-up points) showed that the seizure-free rates were 54.0% (95% CI: 0.43–0.65) and 32% (95% CI: 0.26–0.39) in the primary and conversion monotherapy groups, respectively (Figure 8). The 50% responder rates of PER as monotherapy were not analyzed because only two studies had reported this parameter (Toledano Delgado et al., 2020; Chinvarun, 2022). More patients had experienced ≥50% responder rates in the study by Chinvarun (2022) (patients received PER as primary monotherapy) than those in the study by Toledano Delgado et al. (2020) (most patients received PER as conversion monotherapy) at 3 months (68.3% *vs*. 37.8%, respectively), 6 months (58.5% *vs*. 32.7%, respectively), and 12 months (31.7% *vs*. 24.5%, respectively).

### Impact of enzyme-inducing AEDs on PER efficacy

Some studies have shown that patients who cannot control epilepsy using the initial AEDs could achieve seizure control and even become seizure free after combining AEDs and different MOAs. In general, AEDs can be divided into EIAEDs, which include carbamazepine, oxcarbazepine, phenytoin, and primidone, and non-EIAEDs (any other AEDs). Patients are described as taking EIAEDs if they have received at least one EIAED during their PER treatment. Ten studies including 1,404 patients showed the efficacy of PER with EIAEDs/non-EIAEDs. The results indicated that patients who received combined PER and EIAEDs displayed a slightly low chance of achieving a 50% responder rate. The pooled 50% responder rates were 44.0% (95% CI: 0.28–0.59) and 56% (95% CI: 0.41–0.71) in PER plus EIAEDs and PER plus non-EIAEDs groups, respectively (Supplementary Figure S2). The pooled RR with its corresponding 95% CI for PER plus EIAEDs and PER plus non-EIAEDs groups was calculated. No statistical difference was observed (RR = 0.88, 95% CI: 0.77–1.00), suggesting that the patients treated with combined PER and EIAEDs or non-EIAEDs display similar 50% responder rates. A total of five studies provided data regarding seizure-free rates. The original seizure-free rates were lower in drug-resistant patients with epilepsy (Rinaldi and De Maria, 2018; Lin et al., 2019) than in those who had PER as their first add-on therapy (Santamarina et al., 2020). The pooled seizure-free rates were 17.0% (95% CI: 0.06–0.29) and 25.0% (95% CI: 0.08–0.42) for the PER plus EIAEDs and PER plus non-EIAEDs groups, respectively (Supplementary Figure S3). The estimated RR was 0.61 (95% CI: 0.42–0.91) with low heterogeneity (I^2^ = 30.0%, *p* = 0.22), indicating that patients taking PER with non-EIAEDs had a slightly better chance than those with EIAEDs.

### Clinical safety outcomes

**FIGURE 9:**
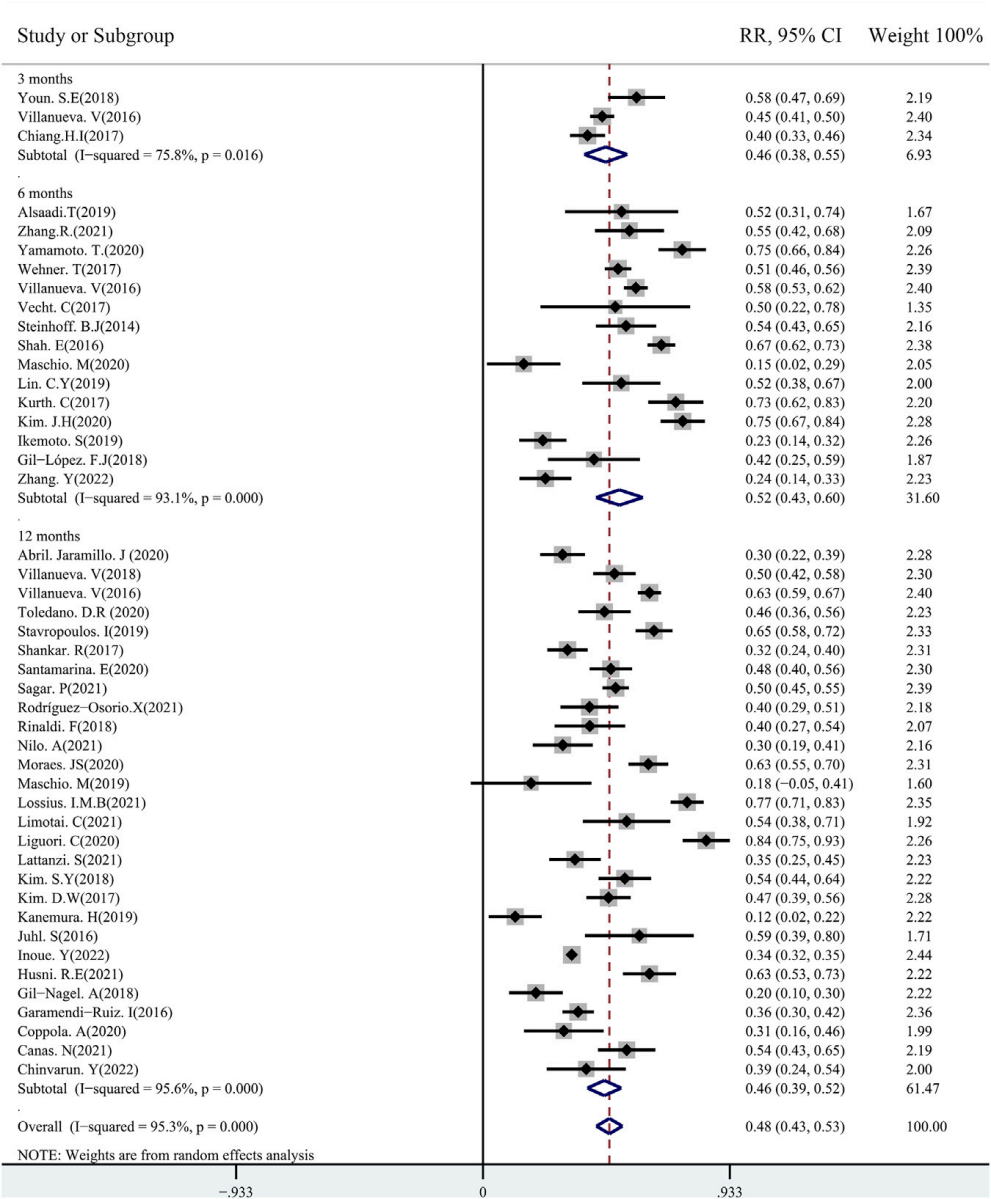
Meta-analysis of adverse events rate: pooled data from 44 studies.

A total of 44 studies (including 8,655 patients) provided data regarding the proportion of patients who experienced at least one of the common treatment-emergent adverse events (TEAEs) after receiving at least one dose of PER. Most adverse events were mild and transient. The results from the random-effects model indicated that the pooled incidences of TEAEs at 3, 6, and 12 months were 46% (95% CI: 0.38–0.55), 52.0% (95% CI: 0.43–0.60), and 46.0% (95% CI: 0.40–0.52), respectively (Figure 9). Notably, nine studies (including 4,548 patients) revealed the proportion of patients who experienced at least one drug-related psychiatric AE after PER treatment. The pooled incidence of drug-related psychiatric AEs was 24% (95% CI: 0.15–0.33). A total of 39 studies (including 7,734 patients) showed the proportion of participants who experienced at least one of the common AEs leading to discontinuation. The pooled withdrawal rates at 3 and 6 months and after 12 months of PER treatment were 8.0% (95% CI: 0.06–0.11), 16.0% (95% CI: 0.13–0.20), and 16% (95% CI: 0.11–0.21), respectively (Supplementary Figure S4). Publication bias was not detected based on the Begg’s test (incidence of adverse events, *p* = 0.70; withdrawal rate, *p* = 0.39).

We further performed a meta-analysis on the 32 AEs (Table 2). In the pooled analysis, the common AEs that were reported in more than 10 studies were dizziness (17%, 95% CI: 0.14–0.20), somnolence (11%, 95% CI: 0.08–0.13), ataxia (6%, 95% CI: 0.04–0.07), headache (3%, 95% CI: 0.02–0.04), cognitive decline/memory problems (2%, 95% CI: 0.01–0.03), sleep disturbance (3%, 95% CI: 0.02–0.04), irritability (9%, 95% CI: 0.06–0.11), aggression (3%, 95% CI: 0.02–0.05), depression (2%, 95% CI: 0.1–0.03), anxiety (2%, 95% CI: 0.01–0.03), fatigue (4%, 95% CI: 0.02–0.06), vision blurred/impairment (13%, 95% CI: 0.00–0.02), and weight gain/loss (2%, 95% CI: 0.02–0.03). It should be noted that other AEs that were reported only in a small number of patients, such as suicidal ideation, rash, and agitation, might also affect tolerability of PER. In addition, one study mentioned some rare AEs, such as limb/joint pain, decreased libido, anguish, face edema, tinnitus, increased blood creatine phosphokinase, influenza, and liver function impairment.

**TABLE 2:** Summary of AEs reported in two or more than two studies in the meta-analysis.

System organ class	Preferred term	No. of studies	Total patients with the AE/total patients treated with PER	Pooled rate (95% CI)
Nervous system disorders				
	Dizziness	33	902/7,015	0.17 (0.14–0.20)
	Somnolence	24	702/6,153	0.11 (0.08–0.13)
	Ataxia/instability	19	152/2,329	0.06 (0.04–0.07)
	Headache	15	84/2,060	0.03 (0.02–0.04), fixed
	Cognitive decline/memory problems	13	57/2,057	0.02 (0.01–0.03)
	Sleep disturbance	10	51/1,510	0.03 (0.02–0.04), fixed
	Dysarthria/slurred speech	4	18/674	0.02 (0.01–0.03), fixed
	Paresthesia/hypoesthesia	3	7/304	0.02 (0.00–0.03), fixed
	Sedation	4	156/718	0.21 (0.16–0.26)
	Fall	3	5/375	0.01 (0.00–0.01), fixed
	Tremor/Parkinsonism	2	3/412	0.00 (−0.01–0.01), fixed
Psychiatric disorders				
	Irritability	22	420/6,142	0.09 (0.06–0.11)
	Aggression	11	106/4,405	0.03 (0.02–0.05)
	Depression	17	79/2,476	0.02 (0.01–0.03)
	Anxiety	10	51/1,443	0.02 (0.02–0.03), fixed
	Confused state/mental slowing	6	19/742	0.02 (0.01–0.03), fixed
	Agitation	5	66/4,054	0.02 (0.01–0.02)
	Psychosis	4	12/819	0.01 (0.00–0.02), fixed
	Suicidal ideation/attempt	4	10/518	0.01 (−0.00–0.03)
	Verbal aggression	2	8/499	0.02 (0.00–0.03), fixed
	Mood change	2	9/252	0.03 (0.01–0.05), fixed
	Hallucinations	2	3/491	0.00 (−0.01–0.01), fixed
General disorders and administration site conditions				
	Fatigue/tiredness	11	80/1,523	0.04 (0.02–0.06)
Gastrointestinal disorders				
	Appetite change	8	28/1,145	0.02 (0.01–0.03), fixed
	Vomiting/nausea	8	33/1,106	0.02 (0.01–0.04), fixed
	Diarrhea	2	3/110	0.03 (−0.01–0.05), fixed
Respiratory, thoracic, and mediastinal disorders				
	Nasopharyngitis	2	16/178	0.09 (−0.02–0.20)
Eye disorders				
	Vision blurred/impairment	10	21/1,231	0.01 (0.00–0.02), fixed
Skin and subcutaneous tissue disorders				
	Rash/pruritus	7	12/1,076	0.01 (0.00–0.01), fixed
Investigations				
	Weight gain/loss	20	89/2,775	0.02 (0.02–0.03), fixed
Laboratory test				
	Gamma-glutamyl transferase increase	2	3/179	0.02 (−0.03–0.037)

Abbreviations: AE, adverse effects; 95% CI, 95% confidence intervals.

### Impact of titration speed on safety of PER

**FIGURE 10:**
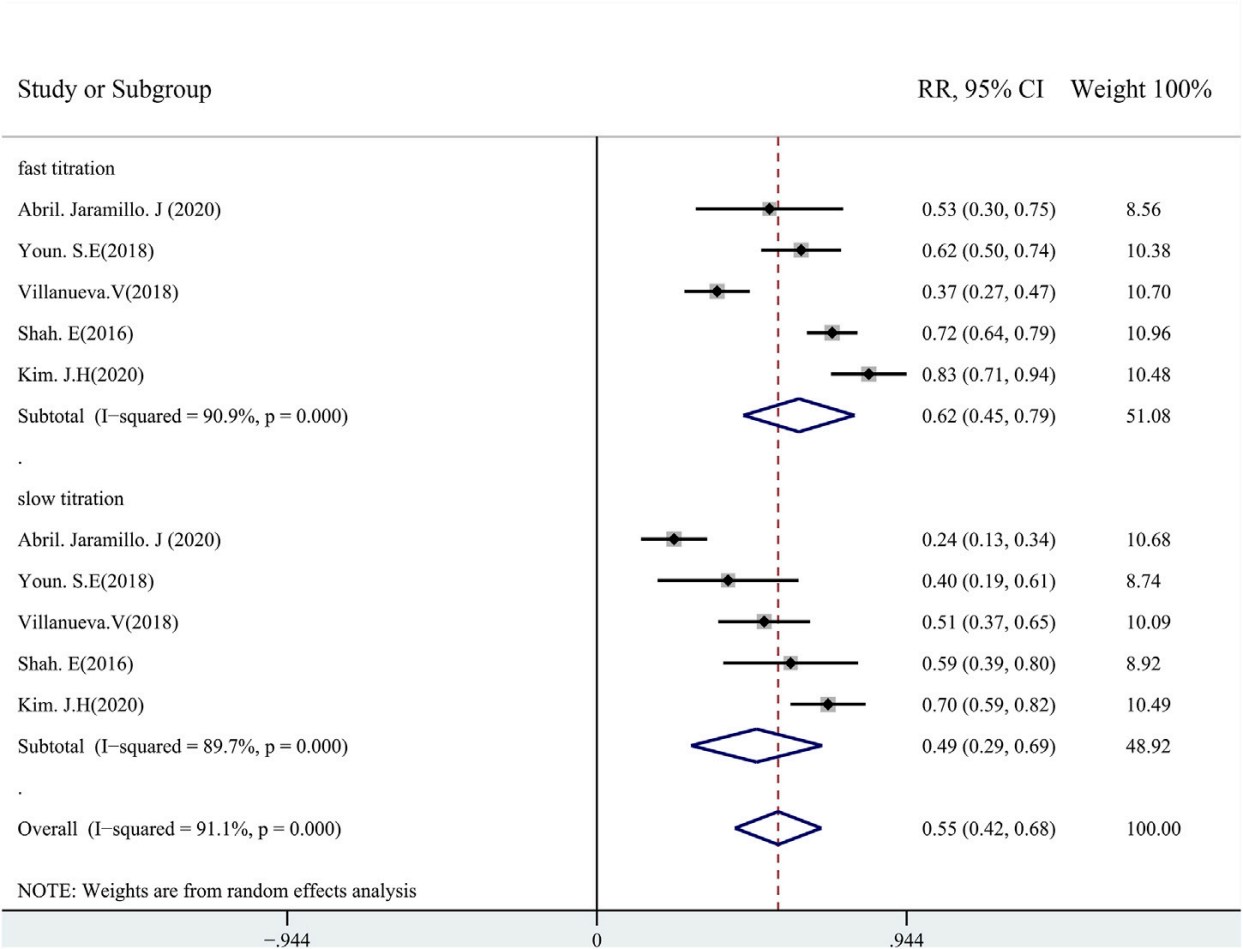
Withdrawal rate due to adverse events (rapid *vs*. slow dose titration).

In routine clinical practice, patients orally received PER tablets starting from 2 mg/day before bedtime. Then, the PER dose was increased from 2 mg/day at intervals of <2 weeks (fast dose titration) or ≥2-week intervals (slow dose titration) up to the desired dose or a maximum of 12 mg/day depending on the clinical outcome. A total of five studies (including 562 patients) revealed the incidences of AEs of PER by using slow or fast titration regimens, in which three studies provided the withdrawal rates due to AEs. There were no significant differences in the pooled retention rates among these studies with rapid (58%, 95% CI: 0.46–0.70) *vs*. slow dose titration (56%, 95% CI: 0.305–0.83) (Supplementary Figure S5). Fewer patients on the slow titration schemes experienced an AE (49%, 95% CI: 0.29–0.69) during the follow-up than those on the fast titration schemes (62%, 95% CI: 0.45–0.79) (Supplementary Figure S6). The pooled withdrawal rates due to the AEs were 30% (95% CI: 0.22–0.38) and 12% (95% CI: 0.06–0.18) in the rapid and slow titration group patients, respectively (Figure 10). The estimated RR was 0.50 (95% CI: 0.28–0.88) with low heterogeneity (I^2^ = 14.1%, *p* = 0.31), indicating that there were significant differences between the groups of slow *vs*. rapid dose titrations.

### Interactions: Enzyme-inducing AEDs on safety of PER

A total of five studies assessed the occurrence of AEs of PER with EIAEDs/non-EIAEDs (Garamendi-Ruiz et al., 2016; Villanueva et al., 2016; Rinaldi and De Maria, 2018; Youn et al., 2018; Santamarina et al., 2020). However, the exact number of patients who experienced AEs was not available in one study (Villanueva et al., 2016). Among the remaining four studies, three studies had reported the proportion of patients with AEs at 12 months and one study had reported the occurrence of AEs at 3 months. In the patients who were receiving PER plus EIAEDs or non-EIAEDs, a similar percentage of AEs during the follow-up was observed. The pooled total incidence of AEs (including all follow-up points) was 35.0% (95% CI: 0.28–0.43) and 37% (95% CI: 0.24–0.49) for patients in the PER plus EIAEDs or non-EIAEDs groups, respectively (Supplementary Figure S7). The withdrawal rates due to AEs were not included because this parameter was analyzed in only two studies (Rinaldi and De Maria, 2018; Santamarina et al., 2020). However, it appeared that more patients taking PER and concomitant EIAEDs discontinued because a high AE was reported in the study by Rinaldi and De Maria (2018) compared with that of Santamarina et al. (2020) (41.9% *vs*. 4.5%, respectively).

### Open-label extension studies

In total, 138 patients were enrolled in Study 207. The retention rate over 1, 2, 3, or 4 years was 64.5%, 47.8%, 37.7%, and 13.4%, respectively, and corresponding 50% responder rates of 28.3%, 24.6%, 18.1%, and 6.5%, respectively. During the entire PER exposure, 93.5% of patients experienced at least one of the AEs after receiving at least one dose of PER. Finally, 12.3% of them discontinued due to AEs. Among the 1,264 patients who completed Study 304, 305, or 306, 1,218 patients continued to the extension Study 307. Similarly, the retention rate reached 73.4%, 55.9%, 35.8%, and 6.4% for patients with 1, 2, 3, and 4 years of PER exposure, respectively, and corresponding 50% responder rates of 34.8%, 30.4%, 21.3%, and 4.1%, respectively. During the entire PER exposure, AEs were reported in 91.3% of patients, and this resulted in 16% of the patients withdrawing from Study 307.

## Discussion

The optimal aim of pharmacotherapy for epilepsy is seizure-free treatment without AEs. The current meta-analysis pooled data from 56 real-world observational studies that were all published in the last 5 years and involved 10,688 patients. Our results showed that PER was effective and safe when used both as add-on treatment and monotherapy in patients with epilepsy aged 12 years and older in routine clinical practice, providing sufficient information for developers and prescribers for PER usage in routine clinical practice.

In the current study, the 50% responder and seizure-free rates were pooled to assess the efficacy of PER. Previous phase III multi-centered RCTs (Trials 304, 305, and 306) showed that PER at 8 mg/day appeared to increase the responder rate when compared with a dosage of 2 or 4 mg/day. The pooled 50% responder rates in our study were similar to those in RCTs (Krauss et al., 2012b; French et al., 2012; French et al., 2013), even in patients with 2-year PER treatment, which indicates that PER was effective for long-term treatment. Complete seizure control is one of the main targets of pharmacotherapy. Interestingly, when compared with those in RCTs, high seizure-free rates were observed in our study. There are various factors that could affect the efficacy of PER. All patients in RCTs were treated with at least two different AEDs prior to PER, indicating that included patients were likely to be drug resistant. However, some patients recruited in our meta-analysis received 0–2 AEDs, which might be one of the reasons that the pooled outcome in seizure freedom was better than that of the RCTs. In addition, patients in real-world observational studies usually used a flexible regimen; clinicians could adjust therapeutic schemes on the basis of patients' epilepsy syndromes and seizure types, whereas those in RCTs were titrated to a fixed dose regimen.

This study recognized several factors that might affect the efficacy of PER. First, the current work demonstrated that PER was more effective when PER was used as a first add-on rather than a second or late add-on treatment. The 50% responder and seizure-free rates were significantly higher in the first add-on group than in the late add-on group. Consistent with our findings, a large pooled observational study from 45 European centers showed that PER, when used as a late add-on treatment, was significantly associated with lower chances of seizure freedom in all logistic regression models (Rohracher et al., 2018). The low efficacy of PER used as a late add-on treatment might be due to severe, refractory epilepsy experienced by patients, which is hard to treat. Second, PER used as primary monotherapy displayed a high seizure control rate in patients with FOS, although both PER primary and conversion monotherapy were effective. For FOS add-on treatment, the recommended maintenance dose range of PER is 8 mg–12 mg/day. However, the efficacy of PER at 4 mg/day has not been fully clarified in previous RCTs. Although the 50% responder rates in patients receiving PER 4 mg/day as adjunctive therapy were slightly high in Study 306 and Study 206, no significant difference was detected in seizure freedom when compared with the placebo (Krauss et al., 2012a; Krauss et al., 2012b). However, four out of the five studies (PER as monotherapy therapy) that were included in our meta-analysis showed that most patients responded favorably to 4 mg/day PER. Since 14 days are required for reaching plasma steady state after a dose increase, we recommend that if patients can obtain satisfactory seizure remission at 4 mg/day of PER, they could remain at this dose for another 4 weeks to determine if it is necessary to increase the dosage. Third, our study assessed the influence of EIAEDs on PER efficacy and showed that patients who received PER plus EIAEDs displayed a slight yet significant low seizure-free rate. However, these results should be confirmed by further trials exploring the use of high-dose PER with EIAEDs. PER is eliminated primarily by hepatic metabolism *via* cytochrome P450 (CYP3A4) (Gidal et al., 2013). The concomitant administration of EIAEDs might shorten the half-life of PER and decrease its concentration, resulting in low clinical efficacy (de Biase et al., 2019). Therefore, a high dose of PER might be required to combine EIAEDs to obtain the same efficacy in seizure control as with PER plus non-EIAEDs. In agreement with our speculation, in the US, the recommended starting dose of PER is 2 mg/day for patients taking non-EIAEDs, whereas 4 mg/day is required for patients taking EIAEDs.

Our study has shown that PER is generally well tolerated. Furthermore, the retention rate at 12 months of PER treatment is still as high as 69%, which is superior to results from previous reports with PER 1-year retention [48% (Rohracher et al., 2018) and 55% (Coyle et al., 2014)]. Moreover, nearly half of the patients had a 2-year exposure to PER, and more than one-third of them had a 3-year exposure in the long-term extension studies, indicating that PER was well tolerated in patients with epilepsy. The AE rates at 3, 6, and 12 months of PER treatment were 46%, 52.0%, and 46.0%, respectively, and the withdrawal rates due to AEs were 8.0%, 16.0%, and 16%, respectively. In general, the AEs were mild to moderate and could be tolerated by most patients. Most AEs appeared at the first 6 months of PER treatment and then disappeared. Only a few additional AEs remained. In agreement with our findings, similar AE rates and withdrawal rates were reported in RCTs (Krauss et al., 2012b; French et al., 2012; French et al., 2013).

Subsequently, we have summarized the 32 AEs that were reported in clinical studies. The most common AEs (reported in more than 10 studies) occurred in the nervous system and displayed psychiatric disorders such as dizziness, somnolence, irritability, ataxia, irritability, aggression, and depression. The current postmarking studies had a similar even lower incidence of AEs than those in RCTs. This difference might be due to the flexible regimen of PER in postmarking studies. Our results showed that patients in the slow titration group exhibited low AE and withdrawal rates in comparison to those in the rapid group. However, similar AE rates were observed in patients who received PER with EIAEDs or non-EIAEDs, indicating that EIAEDs had no significant effect on AEs. It should be noted that some rare AEs were also reported in this study such as suicidal ideation, rash, agitation, Gamma-glutamyl transferase increase, and liver function impairment. Further studies should be done to monitor these rare AEs when PER is used.

Our study included 56 real-world observational studies that had been published in the last 5 years. It is therefore reasonable to assume that the results of the current meta-analysis are reliable in the foreseeable future. However, several potential limitations should be noted: 1) some of our subgroup analyses used a small number of patients, and these results should be further verified by larger trials; 2) another limitation is variation in patient demographic and therapeutic schemes across the studies, which might result in substantial heterogeneity; 3) only one study had performed a comparison of PER efficacy between generalized and partial-onset seizures. Although the results showed that those with generalized epilepsy experienced better efficacy than those with focal epilepsy, more clinical trials and studies are required to clarify this point in the future; 4) the efficacy and safety of PER in different epileptic syndromes were not compared in this study since the related results were lacking. Thus, additional well-designed clinical trials with PER in different epilepsies are required to further provide the potential efficacy and profile of PER for controlling seizures.

## Conclusion

In conclusion, our findings confirm the efficacy and safety of PER usage as an add-on and monotherapy for short-term and long-term treatments in patients with epilepsy aged 12 years and older in routine clinical practice. Considering that the efficacy and safety of PER usage might be affected by various factors, therapeutic schemes of PER should be individualized and adjusted for each patient on the basis of their epilepsy syndrome, seizure type, concomitant AEDs, and so on.

## Data Availability

The original contributions presented in the study are included in the article/Supplementary Material; further inquiries can be directed to the corresponding authors.
