# Supplementary material for: Efficacy and tolerability of perampanel in patients with seizures in real-world clinical practice: A systematic review and meta-analysis
Source: Front Pharmacol. 2023 Mar 28;14:1139514. doi: 10.3389/fphar.2023.1139514 (PMC10086234; doi:10.3389/fphar.2023.1139514)
Supplement: Supplementary file 1 [file Table3.DOCX]

**
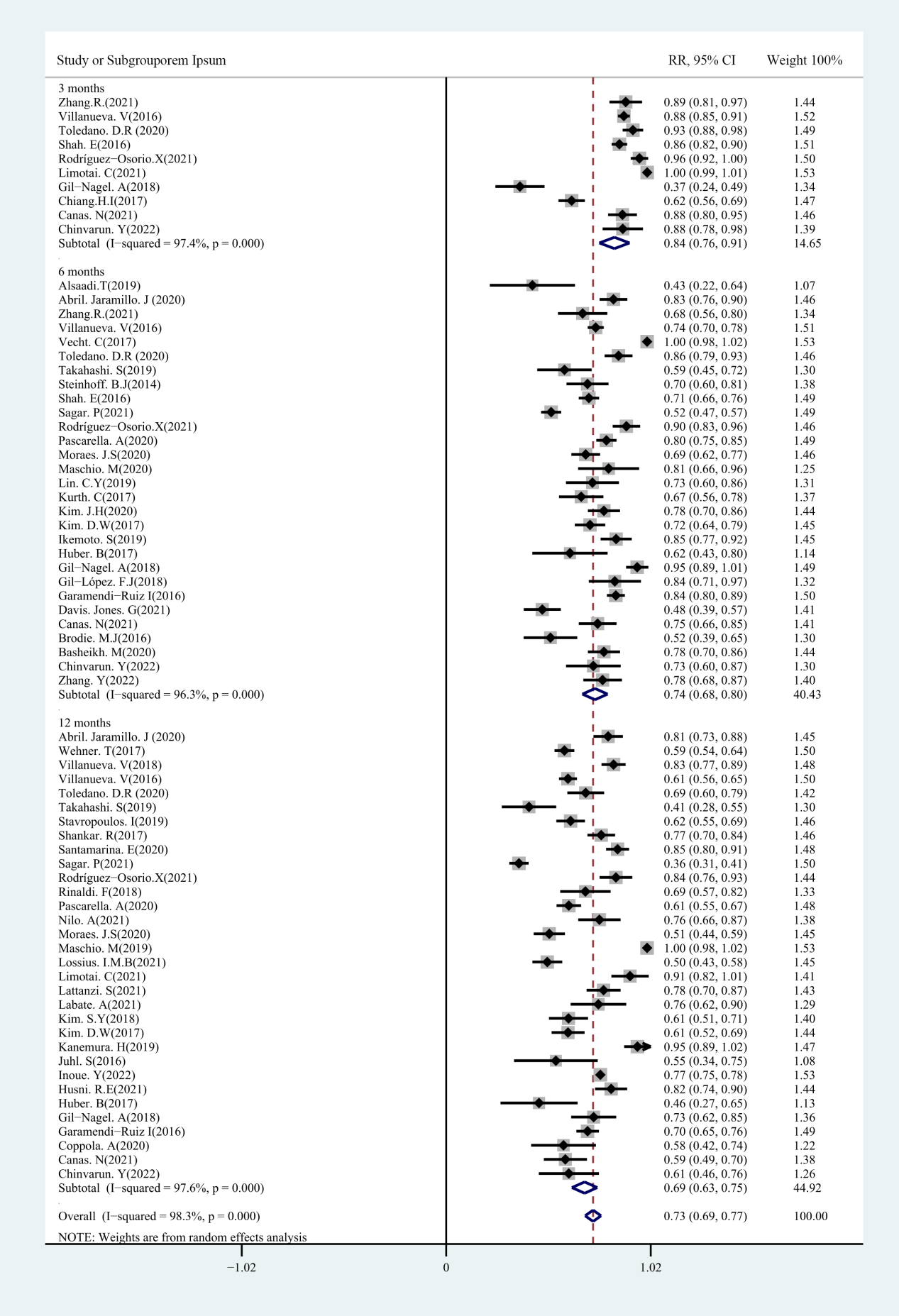
**

**Supplementary Figure 1**. Meta-analysis of retention rate: pooled data from 32 studies


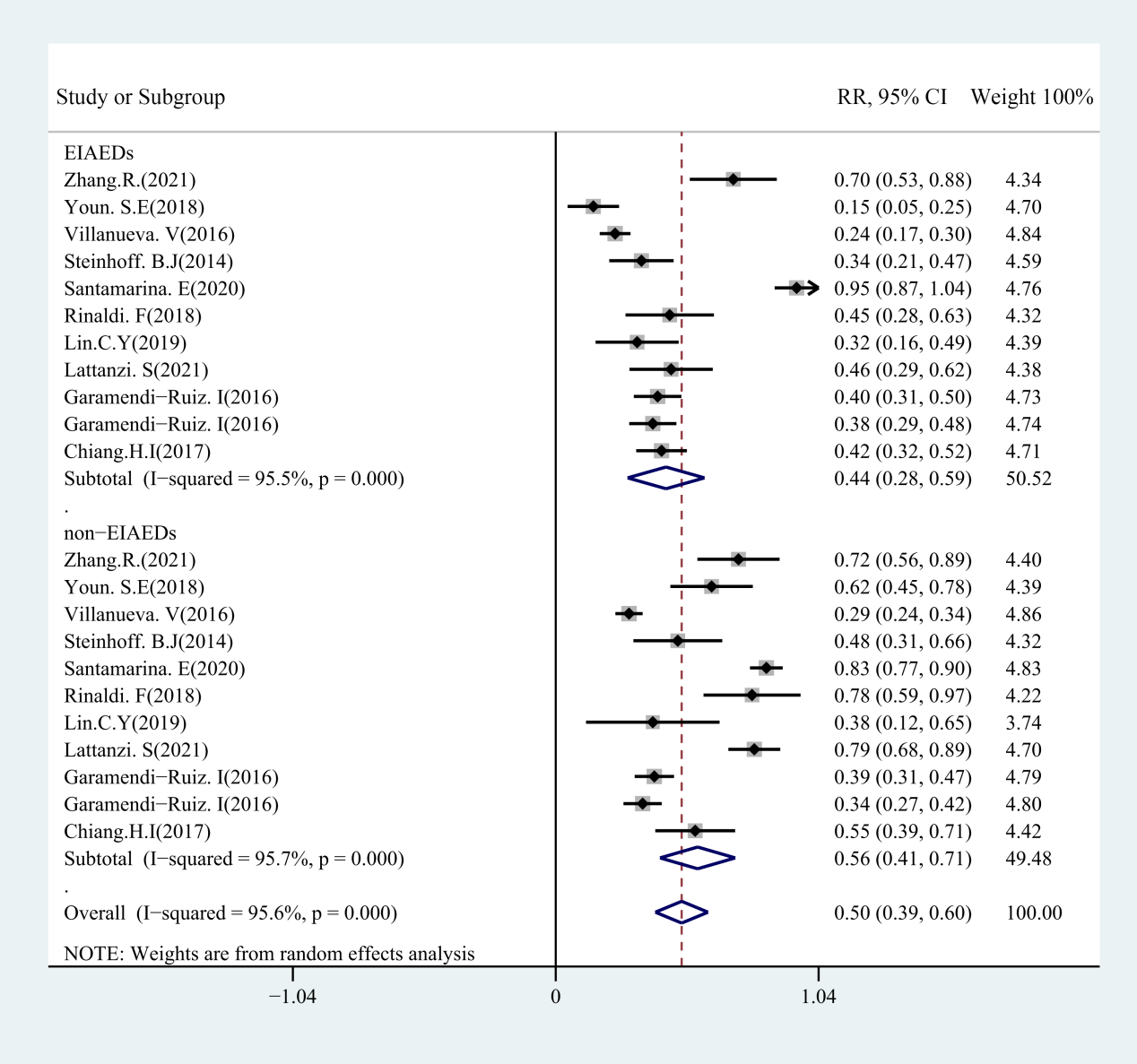


**Supplementary Figure 2**. Pooled 50% responder rate (PER with EIAEDs/non-EIAEDs)

**
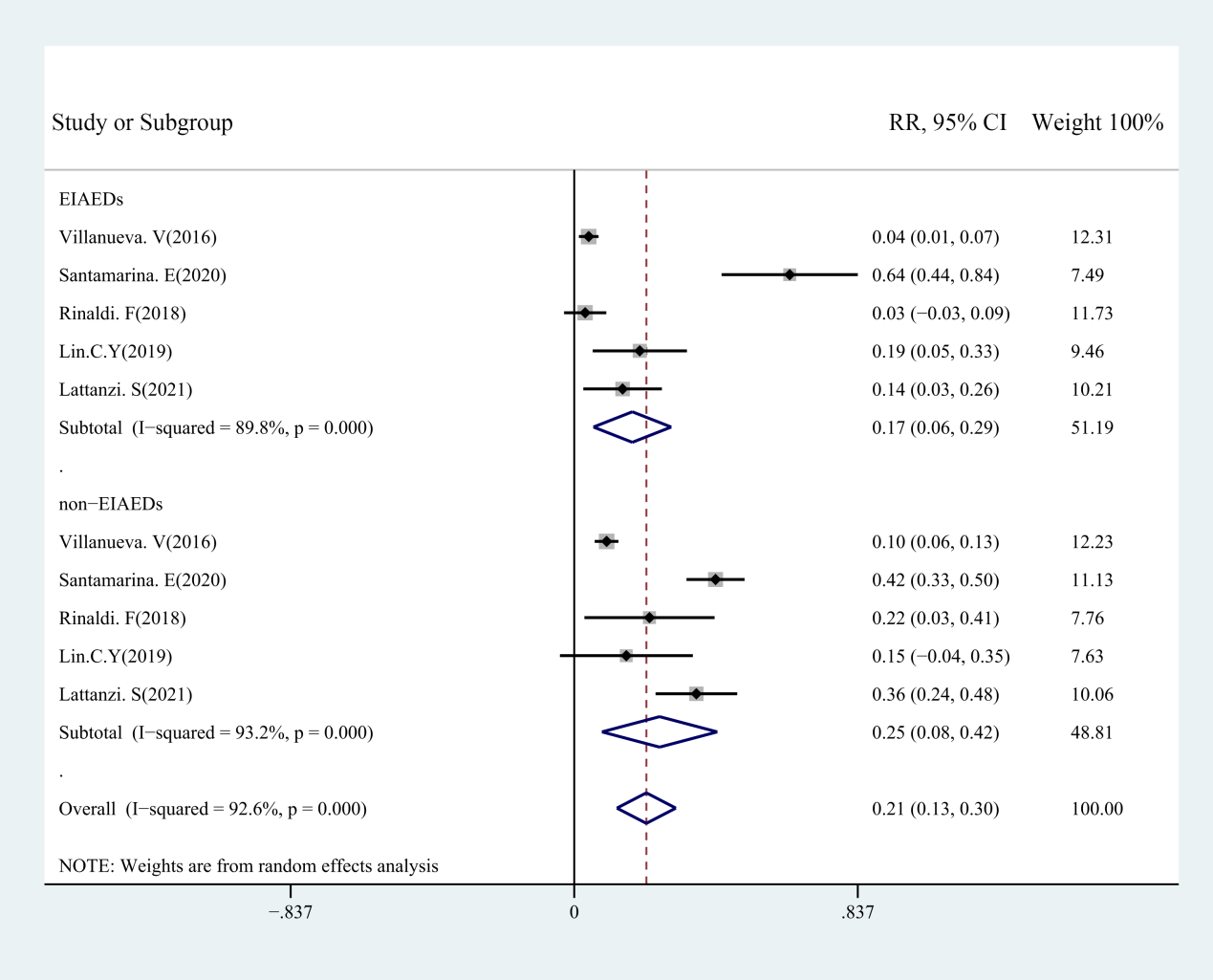
**

**Supplementary Figure 3**. Pooled seizure-free rate (PER with EIAEDs/non-EIAEDs)

**
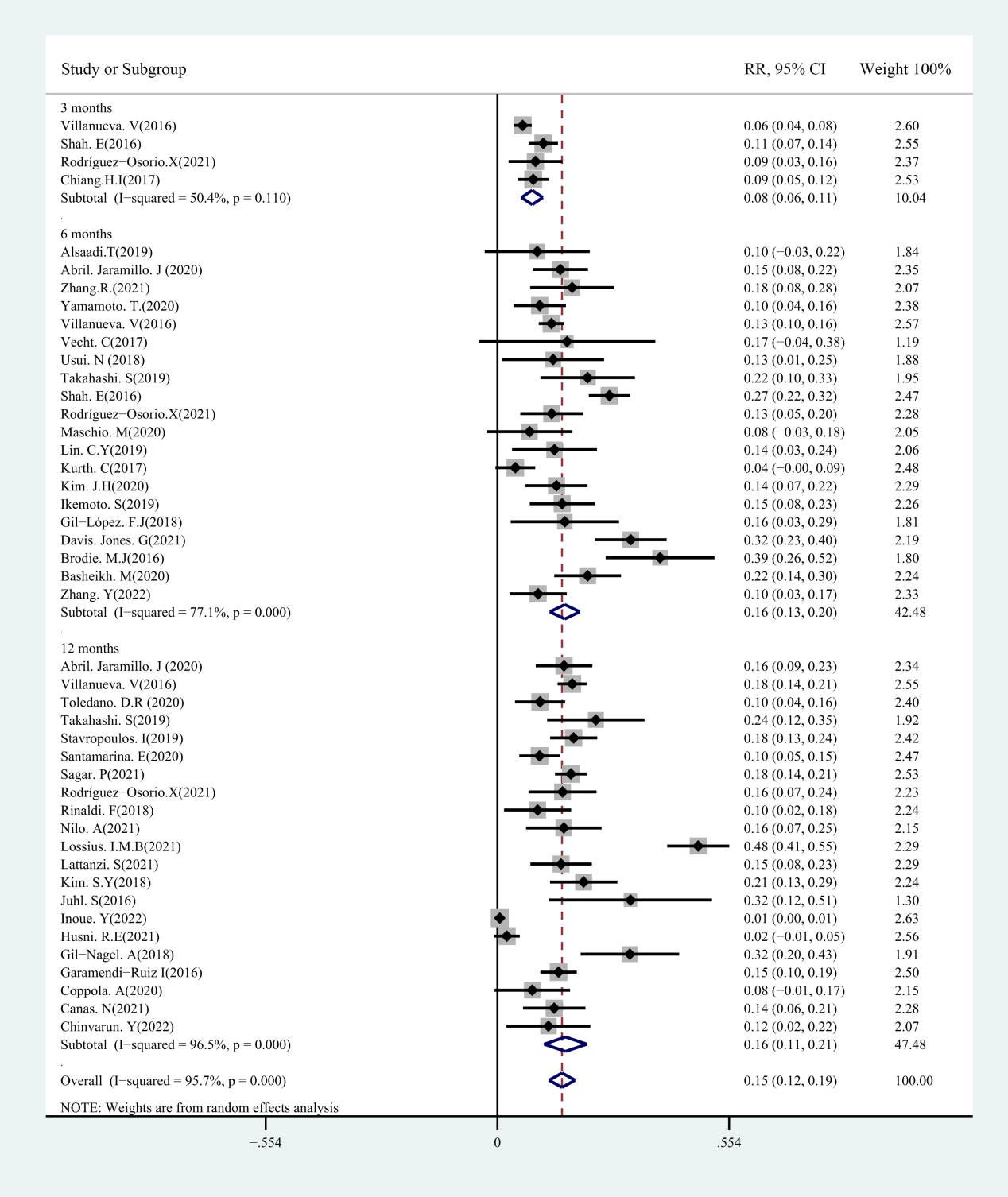
**

**Supplementary Figure 4**. Meta-analysis of withdraw rate due to adverse events: pooled data from 39 studies

**
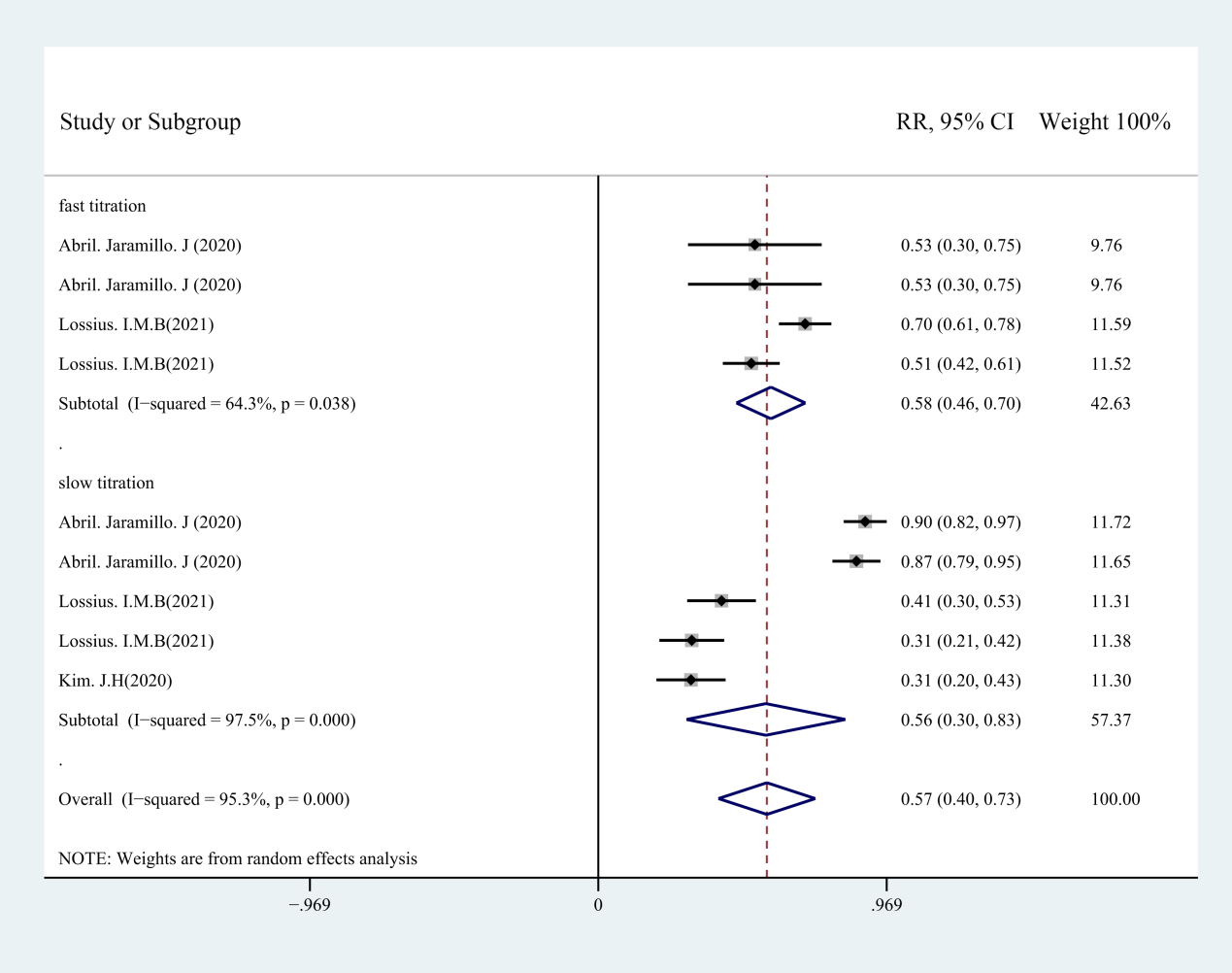
**

**Supplementary Figure 5**. Pooled retention rate (rapid vs. slow dose titration)

**
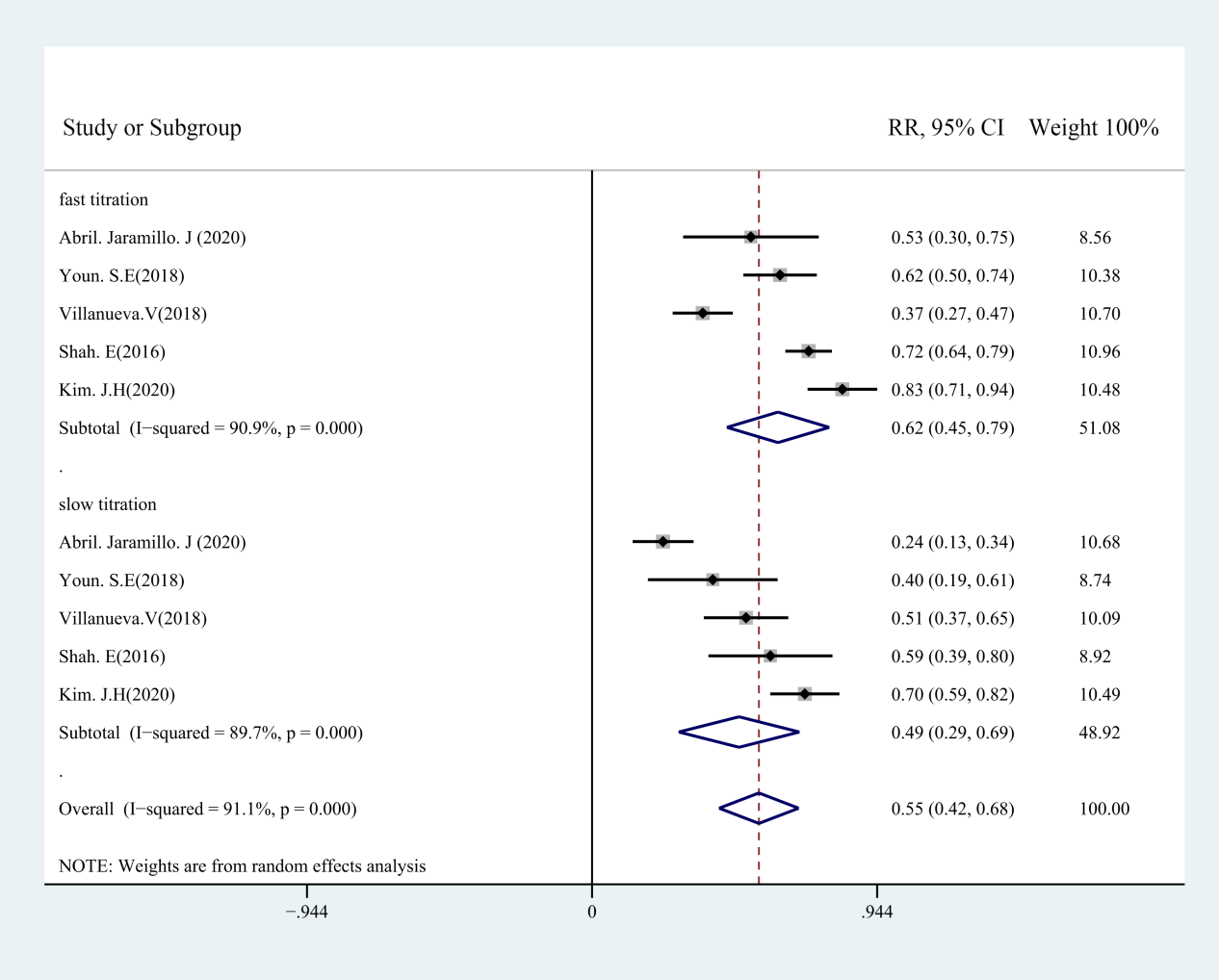
**

**Supplementary Figure 6**. Pooled adverse events rate (rapid vs. slow dose titration)

**
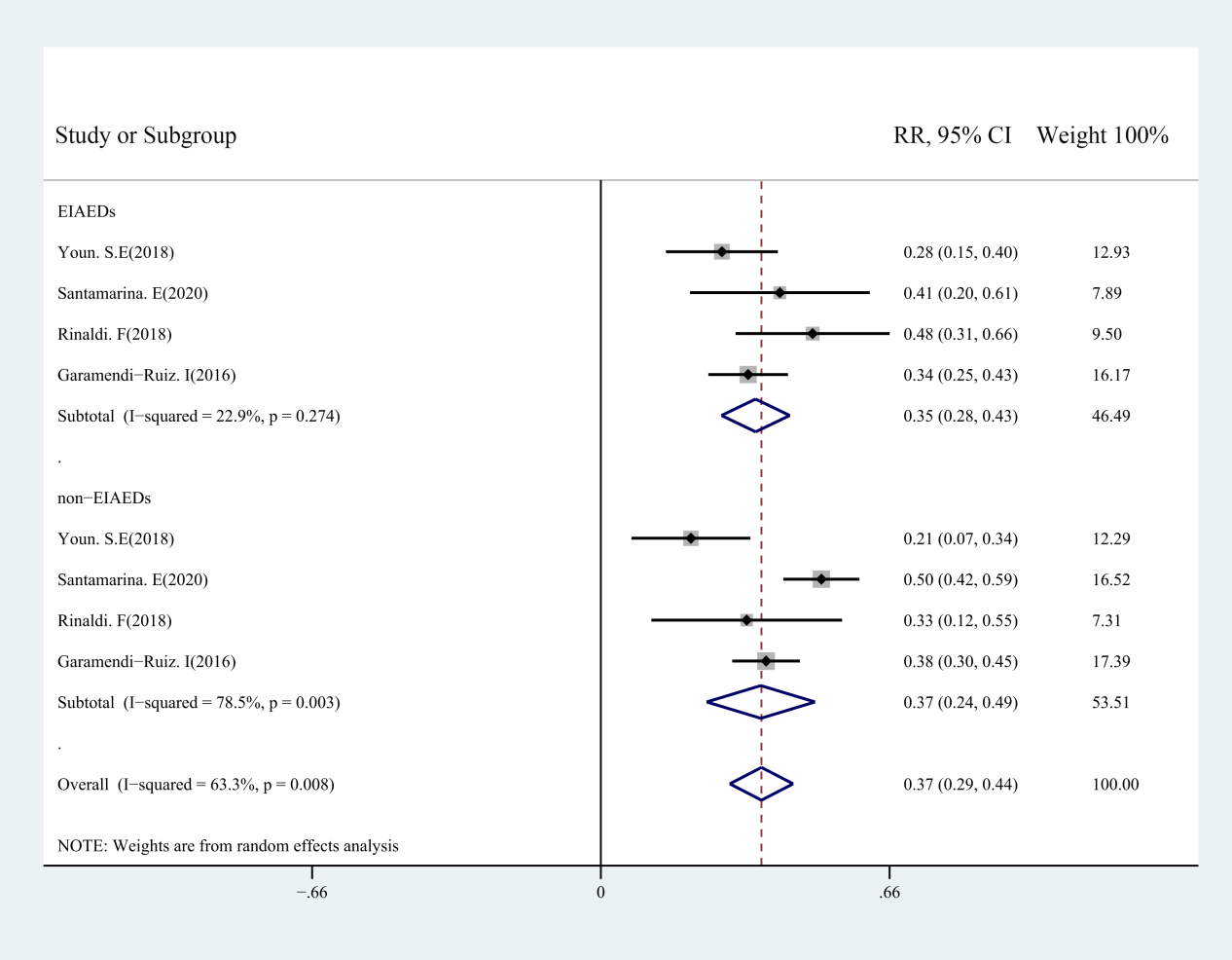
**

**Supplementary Figure** 7. Pooled adverse events rate (PER with EIAEDs/non-EIAEDs)
